# Non-suppressible HIV viremia sustained by clonally expanded CD4+ T cells harboring a genomically defective provirus with an immune-evasive protein expression profile

**DOI:** 10.1128/mbio.03909-25

**Published:** 2026-03-30

**Authors:** F. Harrison Omondi, Yurou Sang, Winnie Dong, Francis Mwimanzi, Peter K. Cheung, Evan Barad, Zerufael Derza, Kieran Anderson, Aniqa Shahid, Vitaliy Mysak, Viviane D. Lima, Mark Hull, Chanson J. Brumme, Marianne Harris, Julio S. G. Montaner, Silvia Guillemi, Mark A. Brockman, Zabrina L. Brumme

**Affiliations:** 1 Faculty of Health Sciences, Simon Fraser University 1763, Burnaby, Canada; 2 British Columbia Centre for Excellence in HIV/AIDS 198129 , Vancouver, Canada; 3 Department of Molecular Biology and Biochemistry, Simon Fraser University 1763, Burnaby, Canada; 4 Department of Medicine, University of British Columbia 8166, Vancouver, Canada; 5 Division of Infectious Diseases, Providence Health Care 102794, Vancouver, Canada; 6 Department of Family Practice, Faculty of Medicine, University of British Columbia 8166, Vancouver, Canada; Duke University School of Medicine, Durham, North Carolina, USA

**Keywords:** human immunodeficiency virus (HIV), non-suppressible viremia, non-subtype B HIV, alternative splicing, clonally expanded provirus, major splice donor (MSD) site, immune-evasive HIV protein expression profile

## Abstract

Antiretroviral therapy (ART) potently inhibits HIV replication but does not eliminate HIV proviruses integrated within the genomes of infected cells. Though ART normally suppresses HIV to clinically undetectable levels in blood, some individuals experience non-suppressible viremia (NSV) that is not attributable to suboptimal drug exposure (e.g*.*, incomplete medication adherence or pharmacological issues) nor emerging HIV drug resistance, and that does not resolve following ART regimen modification. We now understand that NSV can originate from expanded cell clones harboring genetically identical proviruses that reactivate to produce clinically detectable viremia. NSV can even originate from proviruses with genomic defects, particularly within HIV’s major splice donor (MSD) site, which render the viremia non-infectious. But, because only a limited number of such cases have been described, all in HIV subtype B, the mechanisms that allow cells harboring such proviruses to produce prolonged viremia without being eliminated remain incompletely understood. We characterized a case of MSD-defective, replication-impaired NSV that lasted >4 years in an individual with non-B HIV. Our results reveal that proviruses with MSD deletions can persist by integrating into minimally differentiated CD4+ T-cell subsets that then give rise to the full spectrum of memory and effector subpopulations, and by exhibiting an HIV protein expression profile that would allow these cells to evade both cellular and humoral immunity. Our results highlight the need to better understand the biological implications of persistent HIV protein and virion production by clonally expanded cells harboring genomically defective proviruses, and for clinical guidelines to acknowledge this type of viremia.

**IMPORTANCE:**

HIV cure efforts focus on eliminating the viral reservoir, strictly defined as cells harboring proviruses that can produce infectious HIV. But this definition excludes proviruses with defects in HIV’s major splice donor site (MSD), which persist readily. Our results confirm that clonally expanded, MSD-defective proviruses can produce HIV transcripts, proteins, and clinically detectable viremia over long periods, underscoring the need to investigate the biological consequences of these activities. Our findings also have clinical implications. Most HIV treatment guidelines do not acknowledge that persistent viremia during ART can originate from clonally expanded infected cells, and all recommend that viremia >200 HIV copies/mL be managed as virologic failure. Clear clinical frameworks should be developed to discriminate persistent viremia that is due to suboptimal drug exposure and/or emerging drug resistance (that is clinically actionable), from NSV (that is not). HIV MSD genotyping could also help assess viral infectivity, and thus potential transmission risk, during NSV.

## INTRODUCTION

Antiretroviral therapy (ART) halts human immunodeficiency virus (HIV) replication and normally suppresses the virus to clinically undetectable levels in blood. Despite this, some individuals receiving ART experience sustained low-level viremia with no obvious cause (1–5). Such cases raise concerns about incomplete ART adherence, suboptimal drug exposure due to pharmacological issues, emerging drug resistance, and possible virologic failure (6–9), but this phenomenon also occurs in highly adherent individuals and in the absence of new drug resistance mutations (1, 2, 5, 10). In such cases, the viremia is typically not resolvable through ART modification or intensification, hence the term “non-suppressible viremia” (NSV) (4, 11).

HIV, like all retroviruses, persists as an integrated provirus within infected cells, and these “reservoir cells” can clonally expand and reactivate at any time to produce infectious HIV, provided the provirus is genomically intact (12–16). It is for this reason that ART needs to be taken for life. Recently, it has been demonstrated that NSV can originate from expanded cell clones that harbor genetically identical HIV proviruses that reactivate to release sufficient virus to be detectable by conventional assays (3, 4, 17, 18). While ART blocks these viruses from infecting new cells, it neither halts clonal expansion nor eliminates the cells themselves, allowing HIV to be released over long periods.

Notably, NSV is not always infectious. It can sometimes originate from proviruses with defects in the 5′ leader region of the HIV genome, specifically within the major splice donor (MSD) site (HXB2 nucleotide 743) (3, 17), which is used to produce the full complement of spliced viral mRNA transcripts (19–21). Though proviruses with MSD mutations/deletions can produce some viral transcripts, proteins, and even viral particles (3, 22), MSD defects generally alter transcript composition, reduce viral RNA packaging efficiency, and impair virion infectivity (22, 23). Nevertheless, the full impacts of MSD defects on HIV spliced transcript production and protein expression remain incompletely understood, as do the mechanisms that allow clonally expanded cells harboring MSD-defective proviruses to produce viremia for years without being eliminated by the immune system. This is in part because relatively few such cases have been described to date (3, 17, 24), all of which were HIV subtype B infections, where NSV clones generally had sizeable (>20 base) deletions or single point mutations in the MSD.

To better understand this intriguing phenomenon, we longitudinally characterized a case of replication-impaired NSV in an individual with a non-subtype B infection that lasted for >4 years and substantially exceeded the >200 HIV RNA copies/mL threshold that commonly defines virologic failure (6, 7, 25). The viremia in this case was defective due to a novel three-base MSD deletion. We identified the CD4+ T-cell subsets harboring the NSV-causing provirus in blood and characterized intracellular HIV transcription and protein expression profiles to examine how these cells evaded immune detection. Our results reveal that proviruses with MSD deletions can persist by initially integrating into minimally differentiated CD4+ T-cell subsets and by exhibiting an HIV protein expression profile that facilitates immune evasion. Our study highlights the need to better understand the potential biological implications of long-term virus release by clonally expanded infected cells, and for HIV treatment guidelines to acknowledge that persistent viremia >200 HIV RNA copies/mL does not always constitute virologic failure.

## RESULTS

### Participant characteristics and sampling

**Fig 1:**
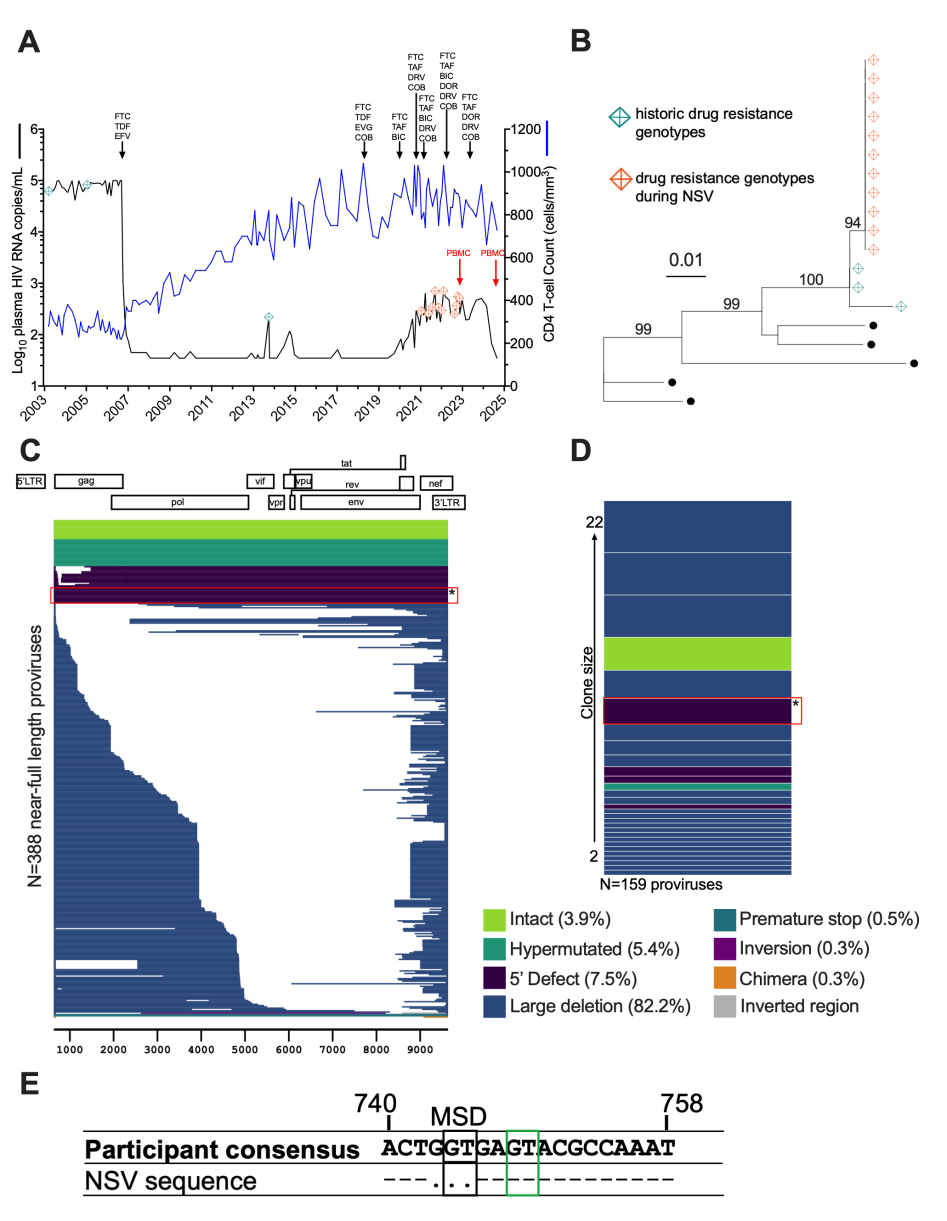
Participant clinical history and sampling timeline. (**A**) Plasma viral load (black line), CD4 count (blue line), and antiretroviral history. FTC = emtricitabine; TDF = tenofovir disoproxil fumarate; EFV = efavirenz; EVG = elvitegravir; COB = cobicistat; TAF = tenofovir alafenamide; BIC = bictegravir; DRV = darunavir; DOR = doravirine. NSV occurred from approximately January 2020 to September 2024. Teal and orange crossed diamonds denote HIV drug resistance testing performed before and during NSV, respectively. Red arrows denote peripheral blood mononuclear cells (PBMC) sampling. (**B**) Maximum likelihood within-host phylogeny of partial *pol* sequences from drug resistance genotypes was performed before and during NSV. Black circles denote reference sequences. Numbers indicate branch support values. Scale is in estimated substitutions per nucleotide site. (**C**) Proviral landscape plot depicting 388 near-full-length proviruses isolated from blood CD4+ T cells during NSV, colored according to genomic integrity (white denotes deletions). The frequencies of each provirus type are shown on the right of the plot. The NSV provirus clone is indicated by the red box with the asterisk. (**D**) Observed provirus clones, colored according to genomic integrity, where bar height corresponds to clone size (numbers to the left of the plot show the range of clone sizes). The clone boxed in red with the asterisk is the NSV provirus. (**E**) Nucleotide alignment of the participant’s proviral consensus sequence versus the NSV sequence in the MSD region, with HXB2 genomic coordinates shown above. The black box denotes the MSD, while the green box denotes a known cryptic splice donor site. Periods denote deletions.

The participant, a Black male in his 50s, was diagnosed with HIV in 2003. However, he believes that he acquired HIV around 1990 (Fig. 1A). By Fall 2006, his CD4+ T-cell count had reached a nadir of 220 cells/mm^3^ and his plasma viral load exceeded 100,000 HIV RNA copies/mL. He initiated ART comprising tenofovir, emtricitabine, and efavirenz. This regimen was maintained until May 2018, when efavirenz was replaced with the integrase strand transfer inhibitor (INSTI) elvitegravir boosted with cobicistat. With the exception of isolated viremia “blips,” plasma viral loads remained suppressed on ART until late 2019, when a viral load of 43 copies/mL was recorded. In early 2020, ART was briefly changed to emtricitabine, tenofovir alafenamide, and the INSTI bictegravir, to simplify to a one-pill-per-day regimen. The viral load measurement 1 month later was 83 copies/mL. A >4-year period of persistent low-level viremia then followed, reaching up to 709 copies/mL, that did not resolve despite multiple regimen modifications, including intensification to four active drug classes. No adherence concerns were noted, and the presence of antiretrovirals in plasma was confirmed using a validated mass spectrometry assay (Table S1) (26, 27). Furthermore, clinical HIV drug resistance genotyping performed 11 times during the viremic period revealed a single unchanging HIV sequence with no drug resistance mutations that was distinct from prior genotypes (Fig. 1B). These observations indicated that the viremia was not due to adherence nor pharmacokinetic issues (9) nor *de novo* HIV drug resistance (8), but likely due to HIV release from a clonally expanded infected cell population (3, 4, 17). We henceforth refer to this as “non-suppressible viremia” (NSV).

### The NSV arose from a clonally expanded infected cell population with a three-base deletion in HIV’s MSD

**Fig 2:**
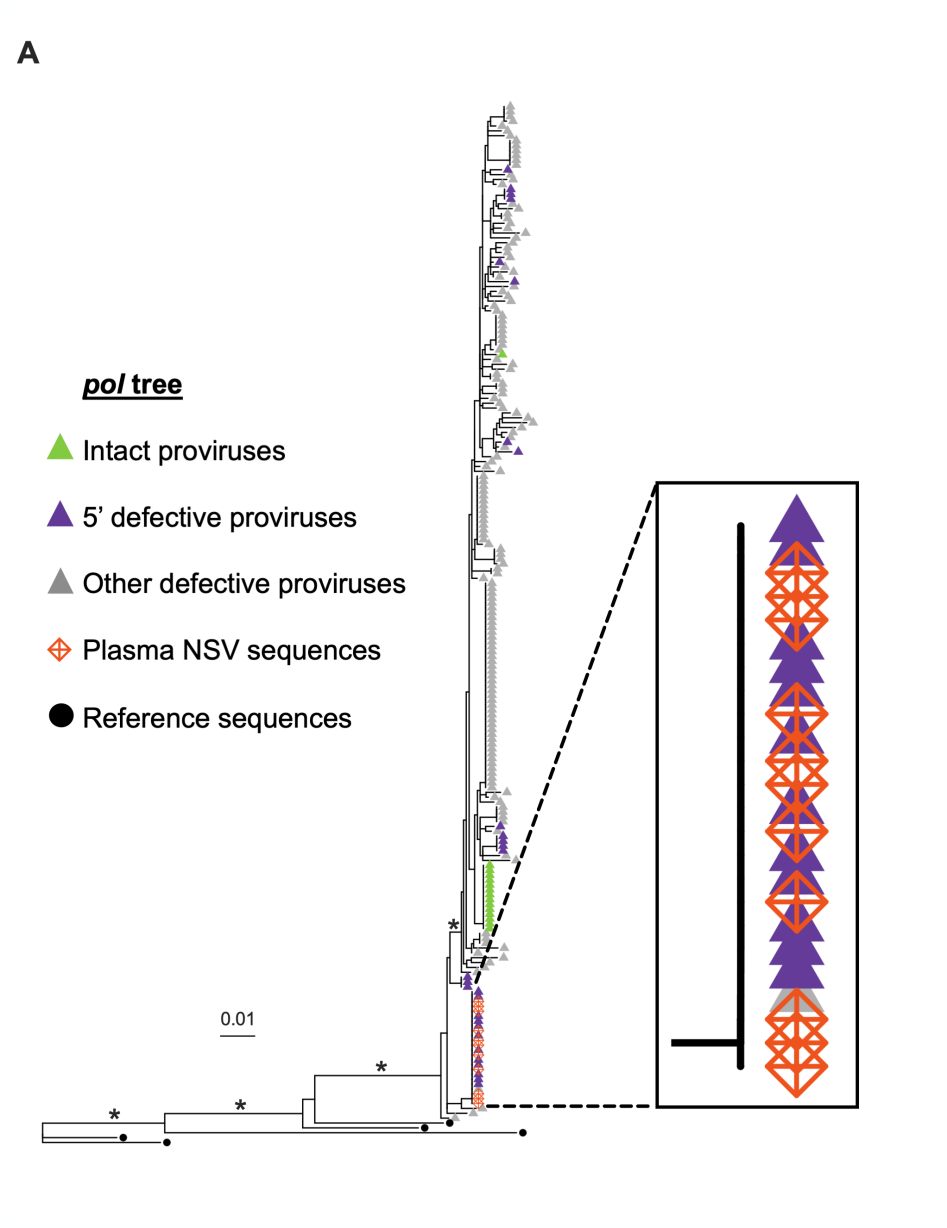
The *pol* sequence of plasma NSV exactly matched a 5′ defective, clonal provirus. (**A**) Maximum likelihood within-host phylogeny inferred from 198 non-hypermutated proviral sequences that contained the partial *pol* region genotyped for HIV drug resistance (where green, purple, and gray triangles indicate intact, 5′ defective, and other defective proviruses, respectively), along with the 11 identical plasma HIV RNA genotypes performed during the NSV (orange crossed diamonds). Black-filled circles denote reference sequences. Asterisks denote branch support values >80%. Scale is in estimated substitutions per nucleotide site.

Approximately 3 years after the NSV began, the participant provided blood from which we isolated 388 near-full-length proviruses from CD4+ T cells by single-genome amplification (Fig. 1C). This revealed the virus was a complex recombinant of subtypes A1, G, CRF01_AE, and CRF02_AG (Fig. S1). Of the proviruses recovered, 3.9% were bioinformatically classified as genetically intact, 7.5% harbored 5′ leader defects, while the remainder had hypermutation, large deletions, or other defects (Fig. 1C). Consistent with clonal expansion, we observed numerous identical proviruses, including a sizeable 5′ defective clone that harbored a three-base “GGT” deletion in HIV’s MSD site that mapped to HXB2 coordinates 743–745 (Fig. 1C and D, boxed in red, and Fig. 1E). The *pol* region of this clone exactly matched the longitudinal drug resistance genotypes that had been performed during the NSV period (Fig. 2). Partial 5′ leader and *env* regions that were single-genome amplified from plasma HIV RNA during the NSV period also exactly matched this provirus sequence (Fig. S2). This indicated that the NSV originated from an expanded CD4+ T-cell clone with a provirus harboring a three-base MSD deletion which was present in the blood.

### The three-base MSD deletion rendered the NSV provirus replication impaired

**Fig 3:**
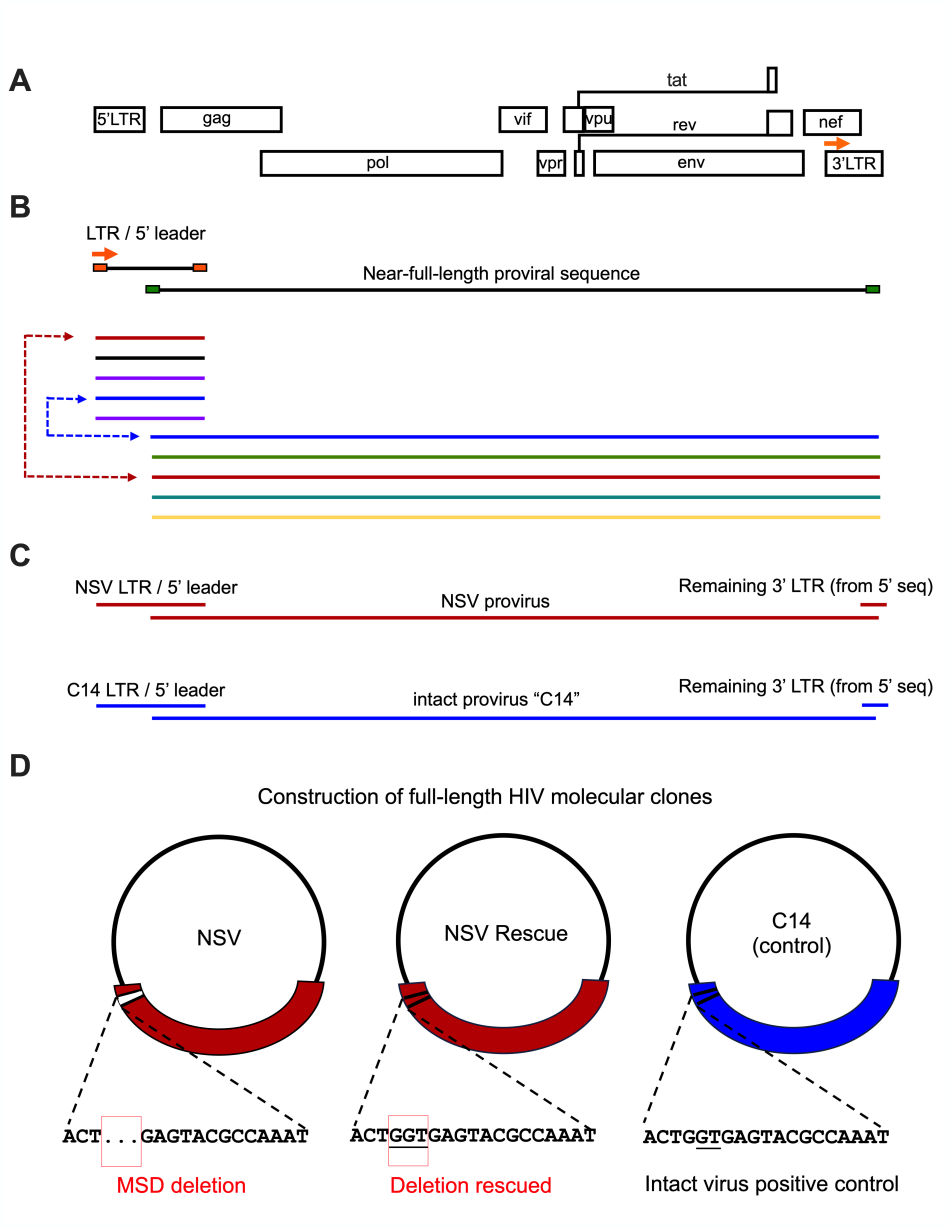
PCR amplification and construction of three autologous HIV molecular clones. (**A, B**) Using autologous primers that bound to the beginning of the participant’s LTR (designed based on the 3′ LTR; orange arrow) and *gag,* respectively, we isolated 81 LTR/*gag* sequences from genomic DNA by single-genome amplification. (**C, D**) The LTR sequence that best matched each near-full-length provirus of interest was used to construct three full-length molecular clones: the NSV provirus (“NSV”), the NSV provirus with the three-base deletion rescued (“NSV rescue”), and another intact provirus from the participant as a control (“C14 control”). The MSD region of each molecular clone is shown below each plasmid.

**Fig 4:**
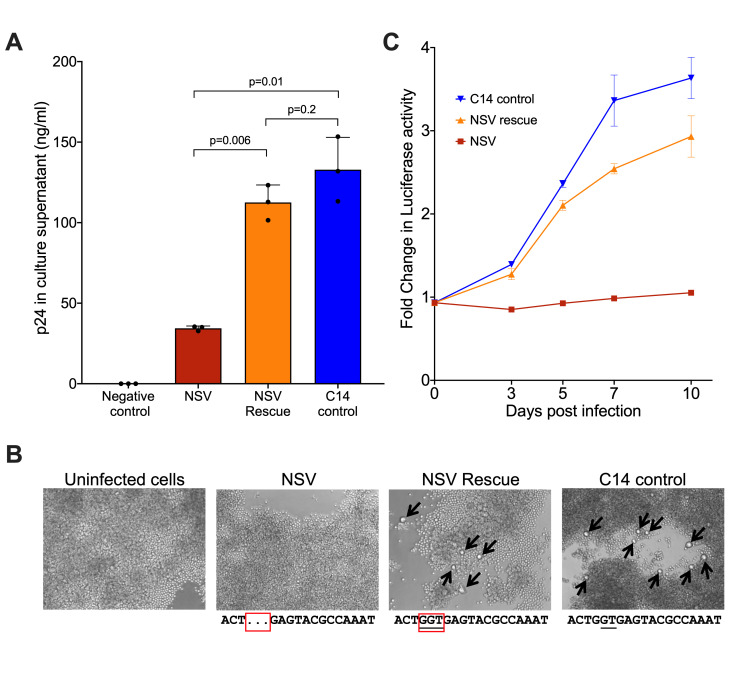
Assessing virion production and replication capacity of the HIV molecular clones. (**A**) The ability of each molecular clone to produce virions was assessed by transfecting equal amounts of plasmid into HEK-293T cells and quantifying p24 in culture supernatants 48 hours later. Bars and whiskers represent the mean and standard deviation of three technical replicates. *P*-values were calculated using an unpaired t-test with Welch’s correction. (**B**) Representative microscopy images showing the formation of syncytia (black arrows) in the NSV-rescue and C14-infected, but not NSV-infected cultures, at 10 days post-infection. The MSD region sequence of each virus is shown below each image. (**C**) Viral spread in culture over 10 days, assessed by quantifying Gaussia luciferase in culture supernatants. Data points and whiskers represent the mean and standard deviation of three technical replicates, respectively.

The three-base MSD deletion suggested that the NSV would be replication impaired. Nevertheless, since most documented MSD deletions are larger (>20 bases) (3, 17, 22) and encompass both the MSD and the cryptic “GT” splice donor site four bases downstream (20), which is preserved in the NSV provirus (Fig. 1E), we investigated the impact of this small deletion in the context of the participant’s unique recombinant virus. As our near-full-length proviral sequences did not capture HIV’s long terminal repeats (LTRs), we single-genome-amplified proviral sequences spanning the 5′ LTR into *gag* from blood CD4+ T cells (Fig. 3A and B) and selected those that best matched our proviruses of interest to construct three full-length autologous molecular clones: the NSV genome harboring the three-base MSD deletion (“NSV”), a modified NSV genome with the MSD deletion repaired (“NSV rescue”) and a representative intact provirus from the participant (“C14 control”) (Fig. 3C and D). For the C14 clone, the selected LTR/*gag* had 100% sequence identity in the region overlapping the near full-length provirus sequence. However, no recovered LTR/*gag* sequence contained the three-base MSD deletion, so we chose the closest match, which differed by eight bases (plus the deletion) in the 587-base overlap region, and re-inserted the MSD deletion. Transfection of HEK-293T cells with equal plasmid copy numbers revealed that the NSV molecular clone produced virions, though to a threefold lesser extent compared to NSV rescue (*P* = 0.006) and C14 control (*P* = 0.01), while virion production from the latter two clones was comparable (*P* = 0.2) (Fig. 4A). This indicated that the three-base MSD deletion reduced, but did not prevent, virion production, which was consistent with the observation of NSV *in vivo*.

We next investigated the NSV clone’s ability to replicate by infecting Sup-GGR reporter cells with equal amounts of NSV, NSV rescue, and C14 virus (inoculum normalized by p24^Gag^ levels). Syncytia were observed in the NSV rescue and C14 cultures after 3 days, while the NSV culture resembled uninfected cells over 10 days (Fig. 4B). Consistent with this, the NSV rescue and C14 cultures showed evidence of viral spread as measured by luciferase production, whereas the NSV culture did not (Fig. 4C). Consistent results were obtained when this experiment was repeated using a co-culture approach to enhance target cell infection, where Sup-GGR cells were added directly to HEK-293T cells that had been transfected with equal amounts of each molecular clone (Fig. S3). This indicates that the MSD deletion conferred a profound replication impairment that could be restored upon deletion repair.

### Identifying the cell type harboring the NSV provirus

**Fig 5:**
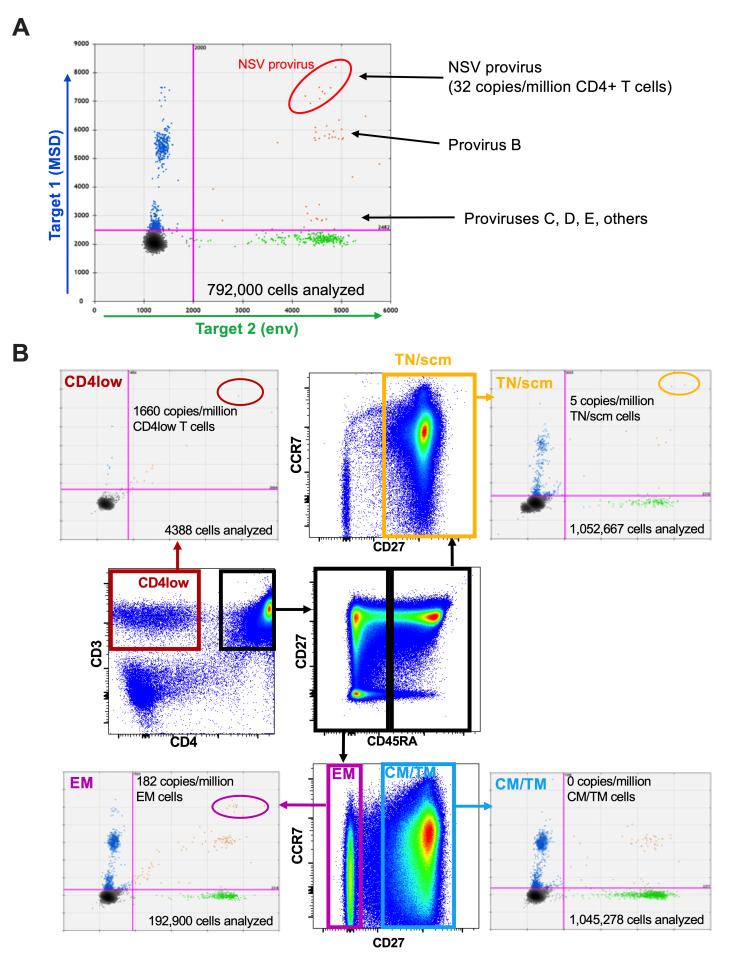
The NSV provirus is found in multiple CD4+ T-cell subsets but is most abundant in effector memory CD4+ T cells. (**A**) Representative ddPCR data from the analysis of bulk genomic DNA from 792,000 CD4+ T cells revealed that the NSV provirus was present at an estimated 32 copies per million total CD4+ T cells. (**B**) The flow cytometry plots in the interior of the figure depict the sorting strategy for blood CD4+ T-cell subsets. Blood CD4+ T cells were isolated by negative selection and then sorted into the following: naïve or stem cell memory cells (TN/scm; CD45RA+CD27+; boxed in yellow in the top flow plot); central or transitional memory cells (CM/TM; CD45RA-CD27+; boxed in blue in the bottom flow plot); effector memory cells (EM; CD45RA-CD27-; boxed in purple in the bottom flow plot); and cells expressing low levels of CD4 (CD4low; boxed in red in the leftmost flow plot). Matching colored arrows point to representative raw ddPCR data from each subset, with estimated NSV provirus copy numbers and total number of cells analyzed shown for each subset.

Notably, the three-base MSD deletion was exclusive to the NSV provirus clone: only two other proviruses harbored it, one that differed from the main clone by only a single base, and the other by only two bases, differences that are within our sequencing assay’s error rate (28). We could therefore exploit this molecular feature to distinguish the NSV from other proviruses. We developed a duplex droplet digital PCR (ddPCR) assay that targeted HIV’s MSD and envelope regions, where the MSD-region probe matched the NSV provirus but was still able to bind, albeit less well, to other autologous proviruses (Fig. S4A). Using synthetic DNA templates, we demonstrated that the MSD probe produced a high-amplitude signal for the NSV sequence and a markedly lower-amplitude signal for other autologous sequences, allowing clear discrimination of the NSV sequence even in mixed templates (Fig. S4B). We further confirmed that the duplexed assay clearly distinguished the NSV provirus from others using our three molecular clones (Fig. S4C). Application of this assay to DNA extracted from 792,000 of the participant’s blood CD4+ T cells revealed that the NSV provirus was present at 32 copies/million CD4+ T cells (Fig. 5A).

We next applied this assay to DNA extracted from different blood CD4+ T-cell subsets to identify those that harbored the NSV provirus (Fig. 5B). Here, we sorted naïve/stem cell memory (TN/SCM) cells (defined as CD45RA+/CD27+), central and transitional memory (CM/TM) cells (CD45RA-/CD27+), and effector memory (EM) cells (CD45RA-/CD27-). Reasoning that the NSV provirus might be expressing functional HIV accessory proteins, including Nef, which can downregulate CD4, we also sorted T cells that displayed reduced surface CD4 expression (“CD4low” subset). We abundantly detected the NSV provirus in the EM subset, at a frequency of 182 copies/million cells. We also detected two events representing the NSV provirus in the TN/SCM-like subset, though none in the CM/TM subset, despite analyzing >1 million cells for each of these subsets. Infrequent detection of the NSV provirus in TN/SCM and CM/TM subsets was confirmed in a separate sort, where we again detected one event in each of these subsets (not shown). We also detected one NSV provirus event in CD4low cells, despite analyzing fewer than 4,400 cells in this subset, indicating that cells harboring the NSV provirus can downregulate CD4. Though the purity of the sorted cells in this specific experiment was not assessed due to the small number of CD4low cells recovered, independent sorts of this participant’s cells yielded ≥98% purity for all sorted subsets, with negligible contamination of CM/TM and TN/SCM with EM cells.

### The NSV provirus exhibited an immune-evasive HIV protein expression profile

**Fig 6:**
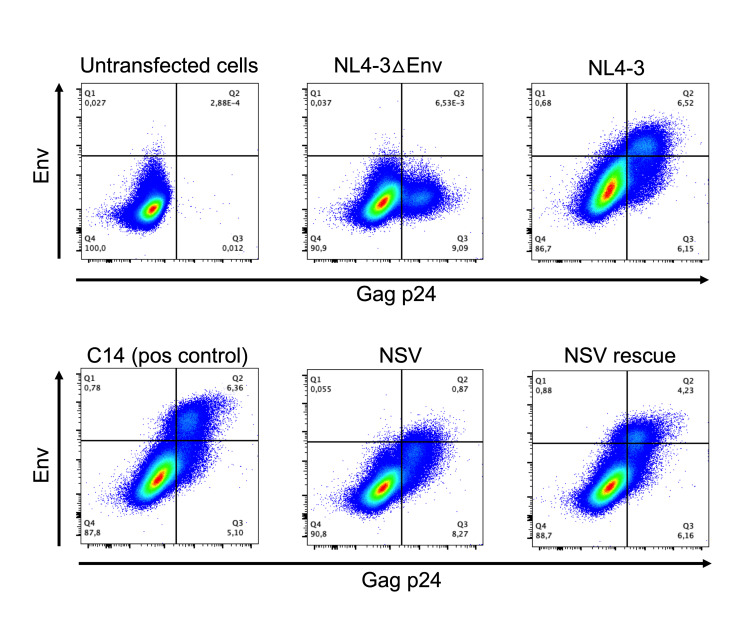
The NSV virus displays impaired envelope expression. Representative flow cytometry plots showing intracellular p24 and cell-surface Env expression in HEK-293T cells 42 hours post-transfection with control HIV molecular clone plasmids (top row), and participant molecular clones (bottom panel). Data from one of two independent experiments are shown.

We hypothesized that the reason the NSV provirus clone had produced viremia for so long without being eliminated was because it exhibited an HIV protein expression profile that allowed it to evade immune detection. Based on prior evidence that HIV subtype B proviruses with 5′ defects produced non-infectious virions with decreased Env incorporation (3), we investigated cell-surface Env in NSV-provirus-expressing cells, reasoning that such defects could explain both impaired virion replication and clone persistence, because reduced Env expression would protect NSV provirus-expressing cells from elimination by antibody-dependent cellular cytotoxicity (ADCC) (29, 30). After transfecting HEK-293T cells with equal copy numbers of the three molecular clones, we observed that only ~10% of the NSV-transfected cells with confirmed p24^Gag^ expression also expressed Env, whereas >40% of the NSV rescue- and C14-transfected cells with confirmed p24^Gag^ expression expressed Env (Fig. 6). Consistent results were obtained when we repeated these experiments in primary cells from an HIV-negative donor, where in this case we infected expanded, CD4-enriched PBMC with equal amounts of VsV-G pseudotyped virus stocks (since primary cells cannot easily be transfected, and the NSV virus’ natural Env makes it poorly infectious) (Fig. S5A). These combined experiments confirmed that Env expression is impaired in the NSV provirus and that this defect is rescued by restoring the MSD deletion.

**Fig 7:**
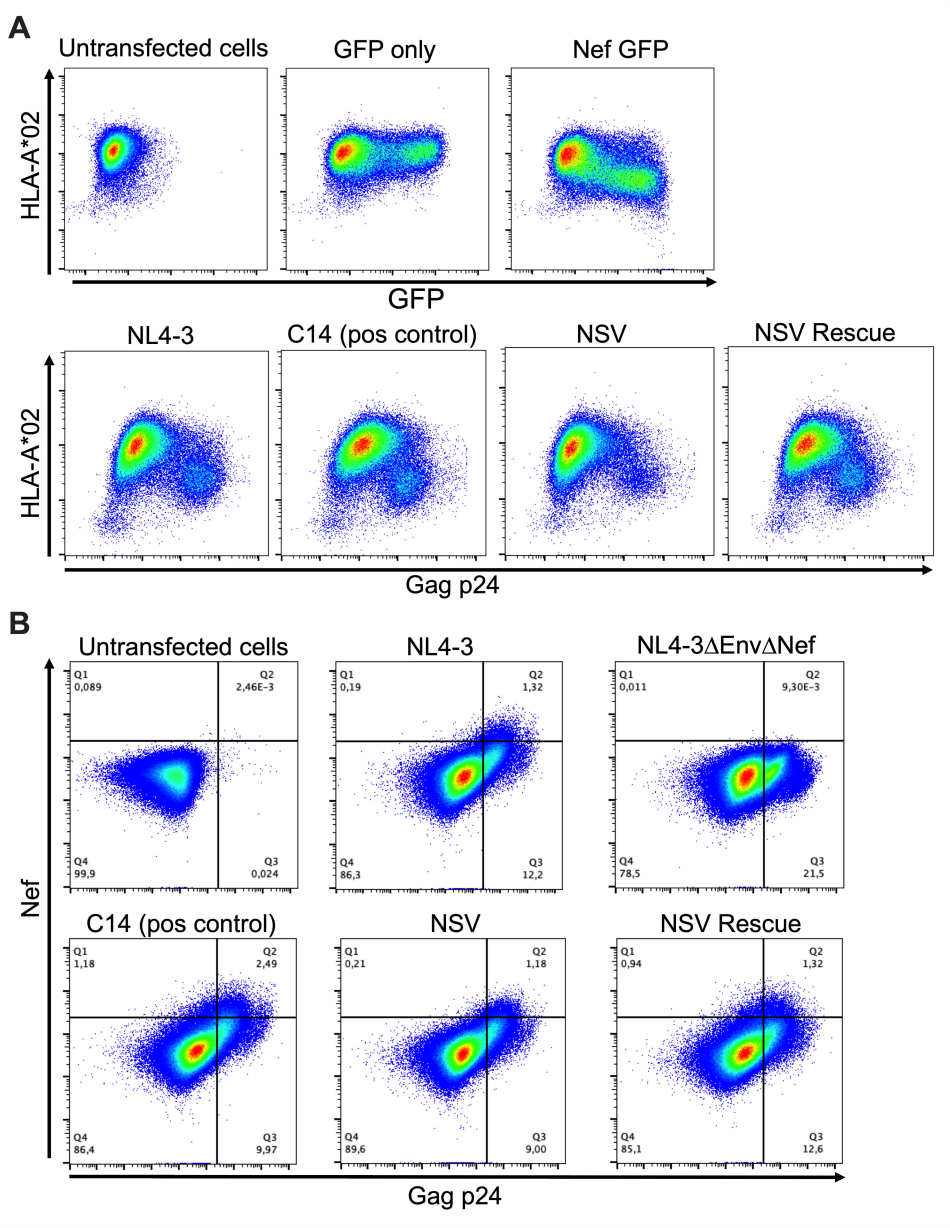
The NSV virus robustly downregulates HLA class I and expresses Nef. (**A**) Top row: Representative flow cytometry plots showing cell-surface HLA-A*02 expression and intracellular GFP expression in HEK-293T cells transfected with control plasmids, including empty pSELECT-GFP (negative control) and pSELECT-GFP expressing *nef* from the HIV subtype B reference strain SF2 (positive control). Bottom row: Representative flow cytometry plots showing cell-surface HLA-A*02 and intracellular p24 expression in HEK-293T cells transfected with a full-length NL4-3 molecular clone (positive control) or participant molecular clones. Data from one of three independent experiments are shown. (**B**) Representative flow cytometry plots showing intracellular Nef expression in HEK-293T cells transfected with control molecular clones (top row) or participant molecular clones (bottom row).

We also investigated the NSV virus’s ability to downregulate HLA class I, which would allow infected cells to evade detection by CD8+ cytotoxic T cells. We transfected HEK-293T cells with equal copy numbers of the NSV, NSV rescue, and C14 molecular clones and assessed HLA-A*02 cell surface expression by flow cytometry (Fig. 7A). All three viruses exhibited similar HLA downregulation abilities, strongly suggesting that, despite its MSD deletion, the NSV virus produces functional Nef. Using the same transfection approach, we confirmed comparable Nef expression by the three molecular clones by flow cytometry (Fig. 7B). The NSV virus’s ability to downregulate HLA class I was also confirmed in primary cells using the VsV-G-pseudotyped virus infection approach described above (Fig. S5B).

Also consistent with the NSV provirus’s preserved HLA downregulation capacity, which would be expected to lessen mutational immune escape pressure on this viral genome, we observed CTL epitopes where one or more intact autologous proviruses exhibited escape mutations, but where the NSV provirus was predicted to retain susceptibility to CTL (the participant’s HLA class I alleles were A*02:02/A*32:01; B*15:03/B*58:02; C*02:10/C*06:02). Within the canonical A*02-restricted epitope spanning Gag codons 77–85 for example, the NSV provirus harbored SLYNTVA**T**L while another intact provirus exhibited the escape form SLYNTVA**V**L (31). Similarly, an epitope spanning RT codons 309–317 that is highly recognized in A*02-expressing individuals (32) was ILKTPVHG**V** in the NSV provirus but ILKTPVHG**T** in other intact proviruses, where the terminal V-to-T was predicted to abrogate HLA-A*02:02 binding. Likewise, the highly recognized HLA-B*57-restricted epitope spanning Vif codons 31–39 (33) (relevant since B*58:02 belongs to the same HLA supertype [34]) was VS**K**KAKRW**F** in the NSV provirus but VS**R**KAKRW**L** in others, where these substitutions were predicted to abrogate B*58:02 binding. These results support the notion that the NSV virus’s ability to downregulate HLA may have lessened pressure for within-host mutational immune escape, at least in some CTL epitopes.

### The NSV virus used two alternative D1 splice donor sites

**Fig 8:**
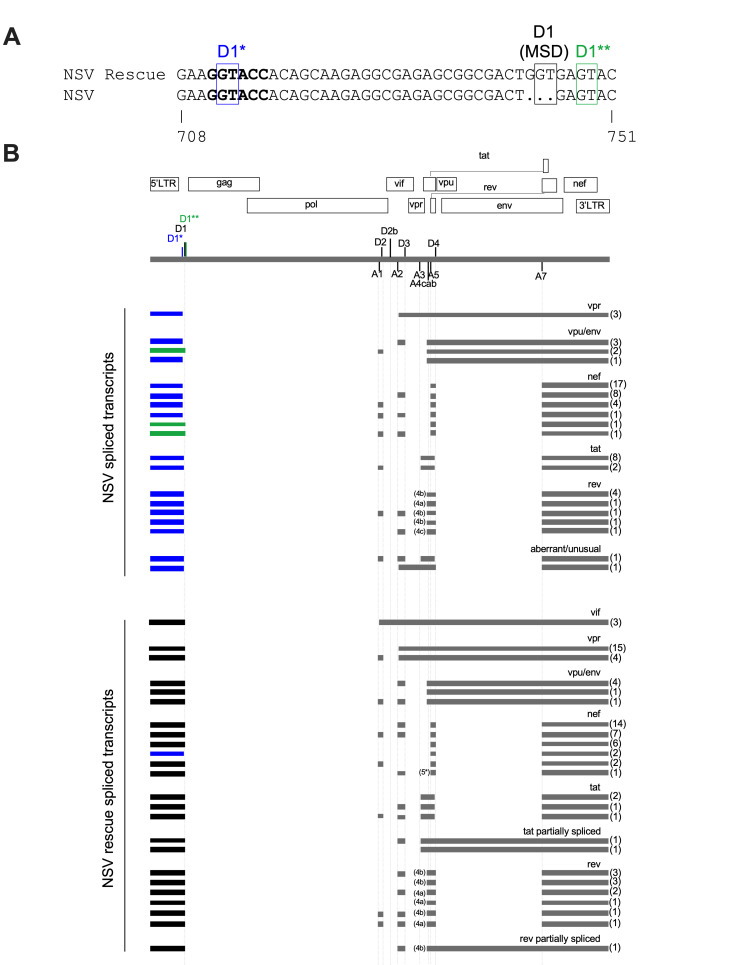
The NSV provirus uses two alternative RNA splice donor sites, and overall spliced transcript composition is altered. (**A**) Nucleotide alignment of NSV and NSV rescue proviruses, showing the MSD (black) and the two alternative splice donor sites used by the NSV provirus: D1* (31 bases upstream; blue) and D1** (four bases downstream; green). HIV’s dimerization initiation site (DIS) is shown in bold. HXB2 genomic coordinates are shown below the alignment. (**B**) Genomic map of the NSV provirus, showing the locations of major splice donor and acceptor sites. Individual spliced HIV transcripts detected in cells transfected with equal amounts of NSV and NSV rescue molecular clones are shown below, where the numbers in parentheses denote how many times each transcript was observed. Exons are depicted as horizontal bars, with the first exon colored based on the D1 site used: MSD (black), D1* (blue), or D1** (green). For the splice acceptor sites 4c, a, and b, the specific site used is shown to the left of this exon. One *nef* transcript observed in NSV rescue-transfected cells used an alternative A5 acceptor site, labeled 5*.

Given that the NSV virus expressed some Nef and a small amount of Env, we investigated which alternative splice donor (D1) site was being used to produce these and potentially other spliced HIV transcripts. Using primers specific for the participant’s autologous virus (Table S2), we single-genome amplified 61 spliced transcripts from HEK-293T cells transfected with the NSV molecular clone. Of these, 57 (93%) had been spliced using a novel cryptic GT motif located 31 bases upstream of the MSD, which we called D1*, while four (7%) had been spliced using a known cryptic GT motif located four bases downstream of the MSD, which we called D1** (3, 20) (Fig. 8A). Of note, D1* fell within HIV’s palindromic Dimerization Initiation Site (DIS) sequence (35, 36), which was GGTACC in this participant’s HIV population (D1* underlined). This DIS sequence differs markedly from the canonical GCGCGC (in subtypes B and D) or GTGCAC (in other subtypes) (37, 38).

In contrast, yet as expected, 76 of 78 (97%) of spliced transcripts from cells transfected with the NSV rescue clone used the MSD site, though two transcripts (3%), both encoding Nef, used the cryptic D1* site within the DIS. Moreover, though NSV-transfected cells produced a variety of spliced transcripts from the 1.8 kb and 4 kb classes, their composition was subtly different from those of NSV rescue-transfected cells (Fig. 8B). First, *vpr*-encoding transcripts were significantly less abundant in NSV-transfected compared to NSV rescue-transfected cells (*P* = 0.0019). Furthermore, *vif*-encoding transcripts were not detected in NSV-transfected cells, though *vif* is typically the least abundant spliced HIV transcript (39, 40). Unusual/aberrant transcripts were also detected in NSV-transfected cells, but not in NSV rescue-transfected cells. Multiply spliced transcripts encoding *tat*, *rev,* and *nef* from NSV-transfected cells also had one fewer exon on average: a median of three, compared to a median of four in cells transfected with the NSV rescue plasmid (*P* = 0.0015).

Most strikingly, for all classes of spliced transcripts, RNA extracts from NSV rescue-transfected cells had to be diluted ~10-fold more than NSV-transfected cell extracts to achieve limiting dilution, even though cultures received the same amount of plasmid and RNA was extracted from the same number of cells. This strongly suggested that the use of alternative D1 sites markedly reduced overall spliced transcript production. Using RT-ddPCR, we confirmed that 1.8 kb-class HIV transcripts were indeed 13 times less abundant in NSV- compared to NSV-rescue-transfected cells (*P* = 0.0006) (Fig. S6). Specifically, in NSV-rescue transfected cells, 1.8 kb-class transcripts outnumbered housekeeping transcripts by a median of 382 (interquartile range [IQR] 361–397)-fold, compared to only a median of 29 (IQR 23–32)-fold in NSV-transfected cells (Fig. S6). Together, these observations indicate that the use of alternative D1 sites markedly reduced spliced transcript abundance and altered their composition, helping to explain some of the defects observed.

### The NSV spontaneously resolved after contraction of the clone population

The participant’s NSV spontaneously resolved after 4.5 years. Analysis of 1.7 million CD4+ T cells shortly after NSV resolution by ddPCR revealed that the NSV provirus population had diminished from the estimated 32 copies/million CD4+ T cells initially measured during the viremic period to an estimated four copies/million CD4+ T cells after viremia resolution, an 8-fold reduction (Fig. S7). By contrast, the total proviral load did not change appreciably over these two time points (1,917 versus 2,161 total proviral copies/million CD4+ T cells, respectively; Fig. S7), nor did the overall proviral landscape as determined by single-genome sequencing (Fig. S8). Notably, no proviruses harboring this unique three-base MSD deletion were isolated by sequencing at the post-NSV time point. Together, these observations are consistent with the NSV resolving upon contraction of the NSV-provirus harboring clonal population.

### Determination of the NSV provirus’s integration site

As a final step, the NSV provirus’s integration site was determined using a modified matched integration site and proviral sequencing (MIP-seq) approach (41). This revealed that the NSV provirus was integrated in the short arm of chromosome 6 p21.1, in intron 1 of the BICRA-like chromatin remodeling complex-associated protein, transcript variant 1 (BICRAL) gene, previously known as the Glioma Tumor Suppressor Candidate Region 1-Like (GLTSCR1L) gene, and originally as KIAA0240 (42, 43). The provirus was integrated in the antisense direction. To our knowledge, HIV integration at this site has not been previously described.

## DISCUSSION

We describe a case of NSV that lasted ~4.5 years in an individual living with a non-B HIV subtype. The viremia was caused by a clonally expanded cell population harboring a provirus with a three-base deletion in HIV’s MSD site, which impaired virus replication. The virus partially compensated for the MSD deletion by using two alternative D1 splice donor sites: one located four bases downstream, previously described in subtype B (3, 20), and another novel site located 31 bases upstream within this individual’s distinctive HIV DIS sequence. These observations highlight the plasticity of splice donor site usage in HIV (21, 44, 45) and underscore the importance of including diverse viral isolates in HIV persistence research.

Despite using alternative D1 sites, the three-base MSD deletion nevertheless markedly impacted spliced HIV transcript abundance and composition, providing insights into the virus’s replication impairment. Consistent with recent NSV cases in HIV subtype B that also featured MSD-deleted proviruses (3), the replication impairment in the present case was attributable, at least in part, to impaired Env expression. NSV-transfected cells exhibited >10-fold reduced spliced transcript abundance compared to NSV rescue-transfected cells (consistent with reduced Env expression on NSV-transfected cells). We also noted that the bicistronic *vpu*/*env*-encoding transcripts in NSV-transfected and NSV rescue-transfected cells differed subtly in their exonic composition: for example, half of the *vpu*/*env*-encoding transcripts in NSV-transfected cells lacked the upstream exon between the A2/D3 splice sites, whereas all but one *vpu*/*env*-encoding transcript in NSV rescue-transfected cells retained this exon, differences that could potentially influence which of the two genes is expressed from the transcript.

Importantly, cells expressing the NSV provirus exhibited an HIV protein expression profile that would have helped these cells evade immune detection despite prolonged viremia production *in vivo*. Though NSV-provirus-expressing cells exhibited markedly reduced spliced transcript levels, they still expressed sufficient functional Nef protein to downregulate cell-surface HLA class I, which would have enabled them to evade CD8+ cytotoxic T-lymphocyte (CTL)-mediated elimination despite HIV protein production (46, 47). Indeed, we identified a number of CTL epitope sequences within the NSV provirus that retained the HLA-susceptible form of the epitope sequence, even though other intact proviruses exhibited escape mutations, consistent with the notion that the NSV virus’s ability to downregulate HLA somewhat lessened pressure to escape through mutation.

At the same time, reduced cell-surface Env levels would protect NSV-provirus-expressing cells from elimination by antibody-dependent cellular cytotoxicity (ADCC). In fact, downregulation of CD4 (e.g., by Nef or Vpu) would have further enhanced the resistance of NSV provirus-expressing cells to ADCC by preventing the required Env-CD4 interaction on the cell surface (48). Our detection of the NSV provirus *ex vivo* in a CD4+ T cell that had downregulated its CD4 receptor also supports this notion and is consistent with a previous study that demonstrated CD4 downregulation in p24-positive cells with 5′ leader defective proviruses (49). On the other hand, the fact that most NSV-provirus-harboring cells expressed normal CD4 levels (as demonstrated by the cell sorting data, which detected CD4 on all NSV-provirus-expressing cells except the aforementioned one) suggested that only a fraction of NSV-harboring clones were actively producing HIV transcripts/proteins at any given time. Viremia production by only a fraction of the expanded clone population is also consistent with previous reports (3, 18). Finally, the observation of impaired *vpr* expression from the NSV provirus is potentially notable. As Vpr arrests cells in the G2/M phase of the cell cycle, thereby preventing host cell proliferation (50, 51), it is conceivable that impaired Vpr expression in the NSV provirus could have contributed to the expansion of this cell clone.

Though HIV can persist in all CD4+ T-cell subsets, including naïve and stem cell memory cells (52–56), proviruses are generally enriched in more differentiated memory subsets, with expanded clones most likely to be effector memory cells (57–59). We detected the NSV provirus in three CD4+ T-cell subsets: at low levels in naive/stem cell memory (SCM) and transitional memory cells (5 and 7 copies per million cells, respectively) and at high levels in effector memory (EM) T-cells (182 copies/million cells). We posit that the NSV virus’s long-term persistence *in vivo* is likely due to its immediate ancestor infecting a T_SCM_, during which the three-base MSD deletion was introduced during reverse transcription (we infer that this initial infection occurred in T_SCM_ because the NSV virus is predicted to use CCR5, which is expressed by T_SCM_ but not naïve T cells). This cell then differentiated into other memory cell types, including the EM subset that was the likely source of prolonged viremia observed here. Though no studies to our knowledge have previously tracked identical proviruses from naive/stem cell memory CD4+ T cells through to more differentiated subsets, previous studies have identified identical proviruses across memory CD4+ T-cell subsets, supporting the notion that clonally expanded T_EM_ can be the progeny of infected T_CM_ cells that proliferate and differentiate (60). Alternatively, it is possible that the NSV-harboring T_EM_ cells that we detected are maintained by central memory cells predominantly located in secondary lymphoid tissues, which could explain why NSV-harboring T_CM/TM_ cells were rare in the blood. After 4.5 years, the NSV-harboring clonal population contracted ~8-fold in size, which coincided with the spontaneous resolution of the NSV, an observation that is consistent with the “waxing and waning” of cells harboring clonal proviruses on years-long timescales (61).

This study has some limitations. The events that triggered the NSV—and its subsequent resolution—are unknown. The clone’s integration site (in the antisense direction into intron 1 of the BICRAL/GLTSCR1L gene on chromosome 6 p21.1) has not, to our knowledge, been linked to integration-site driven clonal expansion (12, 14, 62). That said, BICRAL/GLTSCR1L is a component of the GBAF (GLTSCR1/R1L BRG1/BRM-associated factor) chromatin remodeling subcomplex (63), and it has been shown in a humanized mouse model that HIV integration near genes linked to chromatin regulation is associated with clonal expansion, so we cannot rule out integration site-driven effects (64). Alternatively, it is possible that the NSV was driven by antigen-driven clonal expansion (60, 65, 66) with virus release, and that its resolution was due to clonal contraction upon antigen withdrawal. We were unable to formally test this, however, as we did not determine the antigen specificity of the clone. Of note, a recent report suggested that CD4+ T cells that are reactive to self-antigens can contribute to prolonged NSV, highlighting a potential antigen type that could provide persistent stimulation on a timescale of years (67). Potentially consistent with this, the participant experienced temporary cognitive impairment coincident with NSV onset that did not appear to be explained by virus presence in cerebrospinal fluid (no HIV was detected in CSF collected during this time). An alternative potential mechanism for the clonal contraction could have been terminal exhaustion of the clonal population as a result of persistent high-level antigen stimulation, leading to cell death (68). Alternatively, ART changes could have played a role. A recent paper suggested that changes in HIV therapy—particularly a switch from a non-nucleoside reverse transcriptase inhibitor (NNRTI) to an integrase inhibitor—temporarily perturbed the reservoir in some individuals (69). Of note, the onset of NSV in the present case roughly coincided with a switch to a bictegravir-containing regimen (from an elvitegravir-containing one),where bictegravir was subsequently replaced with the protease inhibitor darunavir before being reinstated (Fig. 1A). The NSV resolved approximately 1 year after bictegravir was replaced with the NNRTI doravirine. Finally, although the NSV virus was profoundly replication impaired *in vitro*, we cannot definitively say it is fully replication-incompetent. In fact, we isolated two proviruses that were near-identical to the NSV provirus (one differed by only a single base; the other by two). Though these mutations are broadly within our assay error estimate of 1.8 × 10^−5^ mutations per base sequenced (28), we cannot rule out that they arose through replication of the MSD-deleted virus *in vivo* prior to ART. Our results also highlight a limitation of some proviral genomic integrity pipelines, which is an inability to identify minor MSD defects. Indeed, the NSV virus required manual reclassification into the 5′ defect category after being initially bioinformatically classified as intact, underscoring the need to improve bioinformatics detection of 5′ defects and the importance of manually verifying all intact sequence calls. Lastly, despite numerous similarities between ours and prior NSV cases described in HIV subtype B (3, 17), our analysis of only a single participant limits generalizability. Larger cohort studies are needed to determine how often NSV occurs in clinical practice. Moreover, additional NSV cases need to be characterized in-depth to determine how often this phenomenon is caused by MSD-defective proviruses, and how genetically and functionally variable these proviruses can be.

HIV cure efforts focus on eliminating reservoir cells, specifically defined as those harboring genetically intact proviruses capable of producing infectious virus. This category excludes proviruses with MSD mutations or deletions, which are assumed to be defective (22, 70–72). The possibility that some proviruses with MSD deletions might be able to replicate (albeit poorly) *in vivo* has potential implications for HIV cure strategies as well as transmission risk during prolonged NSV and underscores the need to understand this class of proviruses better. But even if the *in vivo* replication competence of MSD-defective proviruses is effectively zero, our results add to the growing evidence that they can clonally expand (73), produce viral transcripts (23), HIV proteins (45, 74), and virions over long periods (3, 17). Importantly, our results also indicate that MSD-defective proviruses can exhibit HIV protein expression profiles that could facilitate evasion of both humoral and cellular immune responses, thereby promoting persistence. These observations have negative implications for HIV cure, particularly for “shock-and-kill” reservoir reduction strategies that feature therapeutic latency reversal followed by immune-based clearance (75, 76), as cells harboring such proviruses may resist such treatments. More broadly, our results add to the growing body of evidence suggesting that MSD-defective proviruses are likely to be biologically and clinically relevant. Given that HIV proteins produced by defective proviruses likely drive inflammation (77, 78), and have been associated with clinical outcomes including nonresponse to ART (79), and given the recent demonstration that defective proviruses can conditionally replicate upon superinfection with intact HIV (80), a better understanding of this abundant yet underappreciated proviral class is needed. And, given the unique genetic features of the present case (e.g., three-base MSD deletion; novel splice donor site within a distinctive DIS), this research should include diverse, globally representative HIV isolates.

In conclusion, our findings confirm that NSV can originate from clonally expanded infected cells harboring proviruses with MSD defects (3, 4, 17). Currently, among the major HIV treatment guidelines, only the European ones acknowledge NSV due to cellular proliferation with virus release, but only in the specific context of persistent viremia between 50 and 200 copies/mL in persons fully adherent to ART and in the absence of drug resistance (25). All guidelines continue to recommend that plasma viral loads >200 HIV RNA copies/mL during ART be managed as virologic failure with regimen modification or intensification (6, 7), even though NSV, which can exceed 200 copies/mL as demonstrated by us and others (3, 4, 17), is not resolvable by this action (and in fact, therapy modification would expose individuals to unnecessary drug toxicities, not to mention anxiety when the viremia fails to resolve). Clinical recommendations should be developed to help discriminate persistent viremia that is due to drug adherence, pharmacokinetic issues, or drug resistance development (which is clinically actionable), from NSV (which is not). Presumptive NSV cases could be identified by ruling out suboptimal adherence, pharmacological interactions, and new drug resistance, where the suggested management would be to maintain the current ART regimen, continue to monitor viral loads and CD4 T-cell counts, repeat drug resistance testing at intervals, and if possible, undertake more in-depth HIV RNA sequencing to detect clonality and MSD defects, where the latter would also help assess potential transmission risk during prolonged NSV (81). Indeed, the findings of the present study reassured both participant and care provider that the NSV was not caused by any action (or lack thereof) on their part, allowing the provider to de-intensify ART and alleviate concerns regarding transmission risk during this period.

## MATERIALS AND METHODS

### Amplification and sequencing of plasma HIV RNA

Our clinically accredited laboratory at the BC Centre for Excellence in HIV/AIDS performs HIV drug resistance genotyping for nearly all of Canada, where the genotyped region covers HIV protease and codons 1–400 of reverse transcriptase (82). Briefly, total nucleic acids were extracted from 500 μL of plasma on a NucliSENS EasyMag (bioMerieux), after which bulk cDNA was generated using an HIV-specific reverse primer and NxtScript Reverse Transcriptase (Roche). Subsequent nested PCRs were performed using the Expand High-Fidelity PCR System (Roche). Partial 5′ leader/gag and *gp41* regions were also isolated from plasma HIV RNA by single-genome amplification. For this, nucleic acid extracts were DNase-treated prior to amplification, and cDNA was endpoint-diluted such that subsequent nested PCRs yielded <30% positive amplicons. All PCR primers are listed in Table S2. Amplicons were sequenced using a 3730xl Automated DNA Sequencer (Applied Biosystems), and chromatograms were analyzed using Sequencher v.5.0 (Gene Codes). HIV drug resistance interpretations were performed using the Stanford HIV drug resistance database (83).

### Near-full-length HIV proviral sequencing

Peripheral blood mononuclear cells (PBMC) were isolated from whole blood by standard density gradient separation and cryopreserved at –150°C until use. CD4+ T cells were isolated from PBMC by negative selection (STEMCELL Technologies), and genomic DNA was extracted using the QIAamp DNA Mini kit (Qiagen). Near full-length, single-genome HIV proviral sequencing was performed as previously described (28). Briefly, genomic DNA was diluted such that <30% of resulting nested PCRs, performed using Platinum Taq DNA Polymerase High Fidelity (Invitrogen), yielded an amplicon (Table S2). Amplicons were sequenced on an Illumina MiSeq, and reads were *de novo* assembled using an in-house modification of the Iterative Virus Assembler (84) implemented in the custom software MiCall (version 7.17.0) (http://github.com/cfe-lab/MiCall) to generate a consensus sequence. The genomic integrity of sequenced proviruses was determined using an in-house modification of the open-source software HIVSeqinR (71), where an intact classification required all HIV reading frames, including accessory proteins, to be intact. As no bioinformatics pipeline is perfect, all proviruses initially assigned an intact classification were inspected and manually re-classified where required, with special attention paid to the 5′ leader region. This is because HIVSeqinR defines 5′ defects simply based on sequence length (proviruses with ≥15 base insertions or deletions with respect to HXB2 in this region are considered defective, while all others are classified as intact). Sequences with 100% identity across the entire amplicon were considered identical and clonal. HIV subtyping was performed using the recombinant identification program (RIP) hosted on the Los Alamos HIV sequence database web server using a window size of 400 and a confidence interval of 90% (85).

### Phylogenetic analysis

HIV sequences were codon-aligned using HIVAlign (MAFFT option) (86) hosted on the Los Alamos HIV sequence database (87) and manually edited using AliView (88). Maximum likelihood phylogenies were constructed using IQ-TREE 2 (89) following automated model selection with ModelFinder (90) using an Akaike information criterion (AIC) (91). Phylogenies were visualized using the R package ggtree (92).

### HIV molecular clone construction and replication capacity assessment

We constructed full-length HIV molecular clones in a pUC57Brick vector (GenScript), representing the NSV provirus (“NSV”), the NSV provirus with the three-base MSD deletion rescued (“NSV rescue”) and another representative intact provirus from the participant (“C14”), where the autologous HIV LTR and 5′ leader regions were reconstructed by single-genome amplifying sequences from proviral genomic DNA (primers in Table S2) and selecting the one that best matched each near-full-length provirus in the overlap region (Fig. 3). Molecular clones were propagated in recombinase-deleted Stbl3 *E. coli* (Invitrogen). To generate virus stocks, 13 µg of plasmid was transfected into 2.5 million HEK-293T cells using Lipofectamine LTX (Thermo Fisher Scientific). Supernatants were collected 48 hours later, and p24 levels were measured by ELISA (XpressBio). Virus replication was assessed using the Sup-GGR reporter cell line (93), which contains a Tat/Rev-dependent expression cassette that produces humanized *Renilla* GFP and *Gaussia* luciferase upon HIV infection. For each virus type, we infected one million cells with 13.8 ng of p24. Syncytia formation was monitored by light microscopy, and supernatant *Gaussia* luciferase levels were quantified using a bioluminescence assay (Pierce Gaussia Luciferase Glow, Thermo Scientific) for 10 days. In addition, these experiments were repeated using a co-culture approach, where HEK-293T cells were transfected with 0.125 μg of each molecular clone, and Sup-GGR reporter cells were added to these cultures 24 hours later. Each of these experiments was performed in triplicate, and the results were combined.

### Quantification of the NSV provirus using ddPCR

We designed a dual-target droplet digital PCR (ddPCR) assay modeled upon the Intact Proviral DNA assay (IPDA) (94, 95) to discriminate the NSV provirus from the participant’s other proviruses based on its unique MSD deletion. The assay’s 5′ (MSD) target combined the published IPDA primers with a custom probe that matched the NSV provirus sequence in the MSD region (Table S2 and Fig. S4). The assay’s 3′ (*env*) target used a published secondary *env* probe (95) with autologous primers (Table S2). Reactions were prepared as previously described (94, 95), and data were collected on a QX200 Droplet Reader (BioRad), analyzed using QuantaSoft software (BioRad, version 1.7.4). The 5′ (MSD) target was validated using synthetic DNA templates (IDT gBlocks) spanning HXB2 coordinates 669–919, representing the NSV provirus and the four next most abundant proviruses (Fig. S4). When applying this assay to participant-derived samples, parallel independent reactions to quantify the human RPP30 gene were also performed as previously described for the IPDA, to correct for DNA shearing (94, 95). At least four technical replicates were performed for each ddPCR and merged for analysis.

### Sorting of CD4+ T-cell subsets

We isolated CD4+ T-cell subsets from cryopreserved PBMC as previously described (96) with some modifications. Total CD4+ T cells were labeled with 7-Aminoactinomycin D (AAD) live/dead dye, Peridinin-Chlorophyll-Protein (PerCP)-labeled mouse anti-human CD3 antibody, fluorescein isothiocyanate (FITC)-labeled mouse anti-human CD45RA antibody, and Brilliant Violet (BV421)-labeled mouse anti-human CD27 antibody (all from Biolegend), and allophycocyanin (APC)-labeled mouse anti-human CD4 antibody and phycoerythrin (PE)-Cy7-labeled rat anti-human CCR7 antibody (both from BD Biosciences). Naive/stem cell memory (T_N/SCM_), central memory (T_CM_), transitional memory (T_TM_), and effector memory (T_EM_) CD4^+^ T cells were separated using a FACSAria cell sorter (BD Biosciences) according to the strategy shown in Fig. 5B.

### Flow cytometric analysis of HIV protein expression and HLA downregulation

To measure cell-surface HIV Env expression, we transfected 2.5 million HEK-293T cells with 13µg of NSV, NSV rescue, C14, or control plasmid (NL4-3 or NL4-3Δenv). Forty-two hours later, cells were surface-stained using a cocktail of three primary Env antibodies, 3BNC117 (NIH HIV Reagent program; ARP-12474), VRC01 (ARP-12033), and PGT128 (ARP-13352) (97–100), and PE-labeled anti-human IgG (Biolegend) as secondary. Cells were then stained with Zombie Aqua live/dead dye (Biolegend), fixed/permeabilized (cytofix/cytoperm reagent; BD Biosciences), and intracellularly stained with an APC-labeled anti-p24 antibody (28B7 clone, MediMabs, MM-0289-APC). To measure HLA-A*02 downregulation, we transfected 2.5 million HEK-293T cells with 13 µg of NSV, NSV rescue, or C14 plasmid. A pSELECT GFP-reporter plasmid expressing HIV Nef and an empty pSELECT vector were used as controls. Twenty-six hours later, cells were stained with Zombie Aqua live/dead dye (Biolegend) and APC-labeled anti-HLA-A*02 antibody (BB7.2 clone, Biolegend). After fixation/permeabilization, cells were intracellularly stained with PE-labeled anti-p24 antibody (KC57 clone, Beckman Coulter). To measure intracellular HIV Nef expression, we transfected 2.5 million HEK-293T cells with 13 µg of NSV, NSV rescue, C14, or control plasmid (NL4-3 or NL4-3∆Env∆Nef). Forty-two hours later, cells were stained with Zombie NIR live/dead dye (Biolegend), fixed/permeabilized, and intracellularly stained for Nef using rabbit polyclonal anti-HIV-1 Nef serum as primary (NIH HIV Reagent program, ARP-2949) and BV421-labeled donkey anti-rabbit IgG as secondary (Biolegend). Gag p24 was detected as described above using the APC-labeled 28B7 clone (MediMabs). Flow cytometry was performed on a Cytoflex S instrument (Beckman Coulter). These experiments were performed in triplicate.

HIV protein expression was also assessed in primary cells. Here, VSV-G-pseudotyped stocks of NSV, NSV rescue, C14, and control viruses were produced in HEK-293T cells, concentrated (Total Exosome Isolation reagent, Thermo Fisher Scientific), and p24 levels were measured using p24 ELISA (XpressBio). In parallel, PBMCs from an HIV-negative, HLA-A*02-expressing donor were stimulated with plate-bound anti-CD3 (clone UCHT1) and soluble CD28 (clone CD28.2) antibodies for 3 days, after which CD8+ cells were depleted (EasySep Human CD8-Positive Selection Kit II; STEMCELL Technologies). The resulting CD4-enriched cells were expanded and then re-stimulated with anti-CD3/CD28 for 24 hours, after which 28 ng (p24 equivalent) of each virus stock was used to infect 1 million cells. Sixty-four hours after infection, cell-surface HIV Env expression, HLA-A*02 downregulation, and intracellular HIV Gag expression were measured by flow cytometry as described above, with the following modifications: surface staining featured a BV785-labeled HLA-A2 antibody (clone BB7.2) as well as anti-CD8 staining (FITC, clone SK1) (both from Biolegend) while the intracellular p24 staining featured an APC-labeled anti-p24 antibody (clone 28B7; MediMabs). These experiments were performed in duplicate.

### Characterization and quantification of spliced HIV transcripts

We transfected HEK-293T cells with 13 µg of NSV or NSV rescue plasmid and isolated total cellular RNA 24 hours later (PuroSPIN Total RNA Purification Kit; Luna Nanotech). We generated cDNA using reverse primers designed to capture 4 kb-class, 1.8 kb-class, and universal spliced HIV transcripts and amplified these using single-genome approaches (primers in Table S2). Amplicons were Sanger-sequenced as described above and analyzed in Sequencher v.5.0 (Gene Codes). To quantify the abundance of 1.8 kb-class HIV transcripts, we designed a duplex RT-ddPCR assay that simultaneously measured these, along with a human housekeeping transcript (RPP30). Specificity for the 1.8 kb HIV transcript class was ensured by placing the probe across the D4/A7 splice junction (primers in Table S2).

### HLA class I typing and immune escape predictions

HLA class I typing was performed by independent nested PCR amplification of exons 2 and 3 of HLA-A, -B, and -C loci using primers as described in (101), followed by Sanger sequencing and interpretation using an in-house algorithm. NetMHCpan 4.1 was used to predict binding of epitope sequences to the participant’s HLA class I alleles (102, 103).

### Integration site determination for the NSV provirus

The human genomic integration site of the NSV provirus was determined using a modified matched integration site and proviral sequencing (MIP-seq) approach (41). Genomic DNA extracted from CD4+ T cells was diluted to proviral endpoint in microtiter wells, after which whole human genome amplification was performed by multiple displacement amplification (MDA) using phi29 DNA polymerase and a mixture of Septamer primers and random hexamers (primers in Table S2). MDA products were then subjected to nested PCR (Expand High-Fidelity PCR system; Roche Custom Biotech) that captured the MSD and partial *gag*, and products were Sanger sequenced to identify the wells that contained the unique three-base MSD deletion. These wells were then subjected to a one-round linear extension reaction using two reverse primers located in *gag* downstream of the PstI and SpeI sites that occur naturally in most HIV isolates (primers in Table S2). The integration site was then obtained using inverse PCR (104). Briefly, aliquots of the linear extension products were purified (Ampure XP beads; Beckman Coulter) and digested using either PstI or SpeI (New England Biolabs). Each enzyme cuts frequently in human genomic DNA, but only once in HIV *gag*. Digested DNA was then diluted and self-ligated (T4 DNA Ligase; New England BioLabs). Using two rounds of nested inverse PCR, circularized DNA was amplified outwardly using HIV-specific primers to capture the human genomic sequence in the ligated product (Platinum *Taq* DNA Polymerase High Fidelity, Thermo Fisher Scientific) (primers in Table S2) and Sanger sequenced. The integration site was identified using BLAT (105) hosted on the UCSC genome browser (https://genome.ucsc.edu/cgi-bin/hgBlat). As a final step, the near-full-length NSV provirus was re-amplified from the original genomic DNA sample by nested PCR using forward and reverse primers located in the human and HIV genomes, respectively, followed by sequencing (Illumina MiSeq) as described above, to reconfirm the integration site.

### Statistical analyses

Statistical analyses were performed in Prism (version 8.4.3).

## Data Availability

The participant’s plasma HIV RNA sequences have been deposited in GenBank under accession numbers PX459522-PX459551. Intact proviruses are deposited under GenBank accession numbers PX455285-PX455299, while defective proviruses are deposited under GenBank accession numbers PX487311-PX487683.

## SUPPLEMENTAL MATERIAL

Supplemental figures mbio.03909-25-s0001.pdf Figures S1 to S8.

Table S1 mbio.03909-25-s0002.pdf Plasma antiretroviral levels measured during nonsuppressible viremia.

Table S2 mbio.03909-25-s0003.pdf Primers and probes.

ASM does not own the copyrights to Supplemental Material that may be linked to, or accessed through, an article. The authors have granted ASM a non-exclusive, world-wide license to publish the Supplemental Material files. Please contact the corresponding author directly for reuse.
