# Supplementary material for: Non-suppressible HIV viremia sustained by clonally expanded CD4+ T cells harboring a genomically defective provirus with an immune-evasive protein expression profile
Source: mBio. 2026 Mar 30;17(5):e03909-25. doi: 10.1128/mbio.03909-25 (PMC13170330; doi:10.1128/mbio.03909-25)
Supplement: Supplemental figures — Figures S1 to S8. [file mbio.03909-25-s0001.pdf]

Supplementary Material for:

# Non-suppressible HIV viremia sustained by clonally expanded CD4+ T-cells harboring a genomically defective provirus with an immune-evasive protein expression profile

F. Harrison Omondi[a,b], Yurou Sang[a], Winnie Dong[b], Francis Mwimanzi[a], Peter K. Cheung[a,b], Evan Barad[a,b], Zerufael Derza[a], Kieran Anderson[e], Aniqa Shahid[a,b], Vitaliy Mysak[b], Viviane D. Lima[b,c], Mark Hull [c,f], Chanson J. Brumme[b,c], Marianne Harris[b,d], Julio S.G. Montaner[b,c], Silvia Guillemi[b,d], Mark A. Brockman[a,b,e], Zabrina L. Brumme[a,b#]

[a] Faculty of Health Sciences, Simon Fraser University, Burnaby, Canada
[b] British Columbia Centre for Excellence in HIV/AIDS, Vancouver, Canada
[c] Department of Medicine, University of British Columbia, Vancouver, Canada
[d] Department of Family Practice, Faculty of Medicine, University of British Columbia, Vancouver, Canada
[e] Department of Molecular Biology and Biochemistry, Simon Fraser University, Burnaby, Canada
[f] Division of Infectious Diseases, Providence Health Care, Vancouver, Canada

#Address correspondence to:
Zabrina L. Brumme
Faculty of Health Sciences
Simon Fraser University
8888 University Drive
Burnaby, BC, Canada
zbrumme@sfu.ca

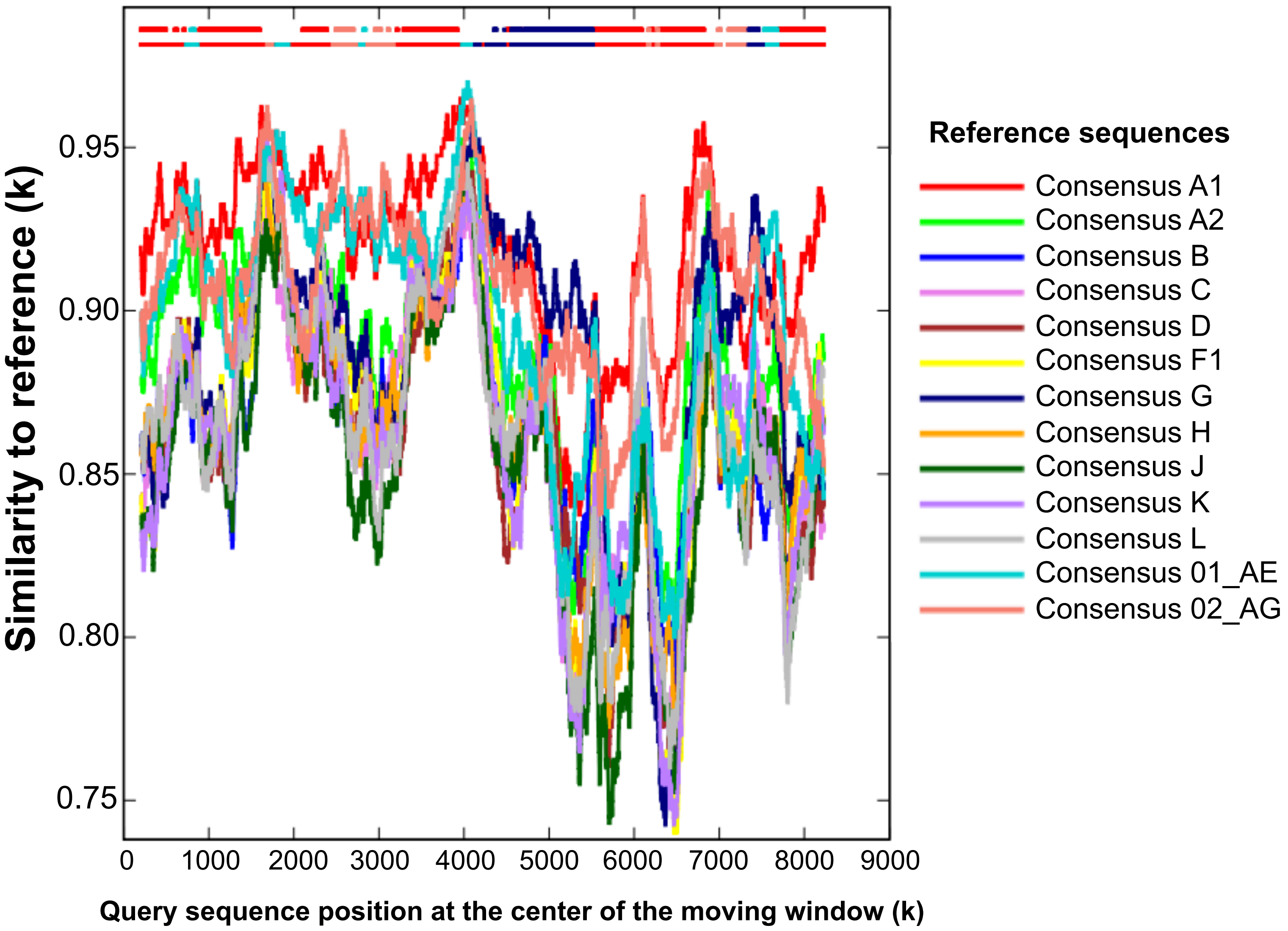


**Figure S1. The participant's HIV is a novel recombinant comprising subtypes A1, G, CRF01_AE, and CRF02_AG.** Recombinant Identification Program (RIP) plot of the full genome sequence of a representative provirus isolated from the participant. The y-axis denotes the % similarity between the participant and each of the 13 reference sequences (each shown by a different color), using a sliding window of 1000 bases. The two lines at the top indicate the best matching reference sequence over a given region (lower bar) and whether this match meets the 90% confidence threshold (upper bar).

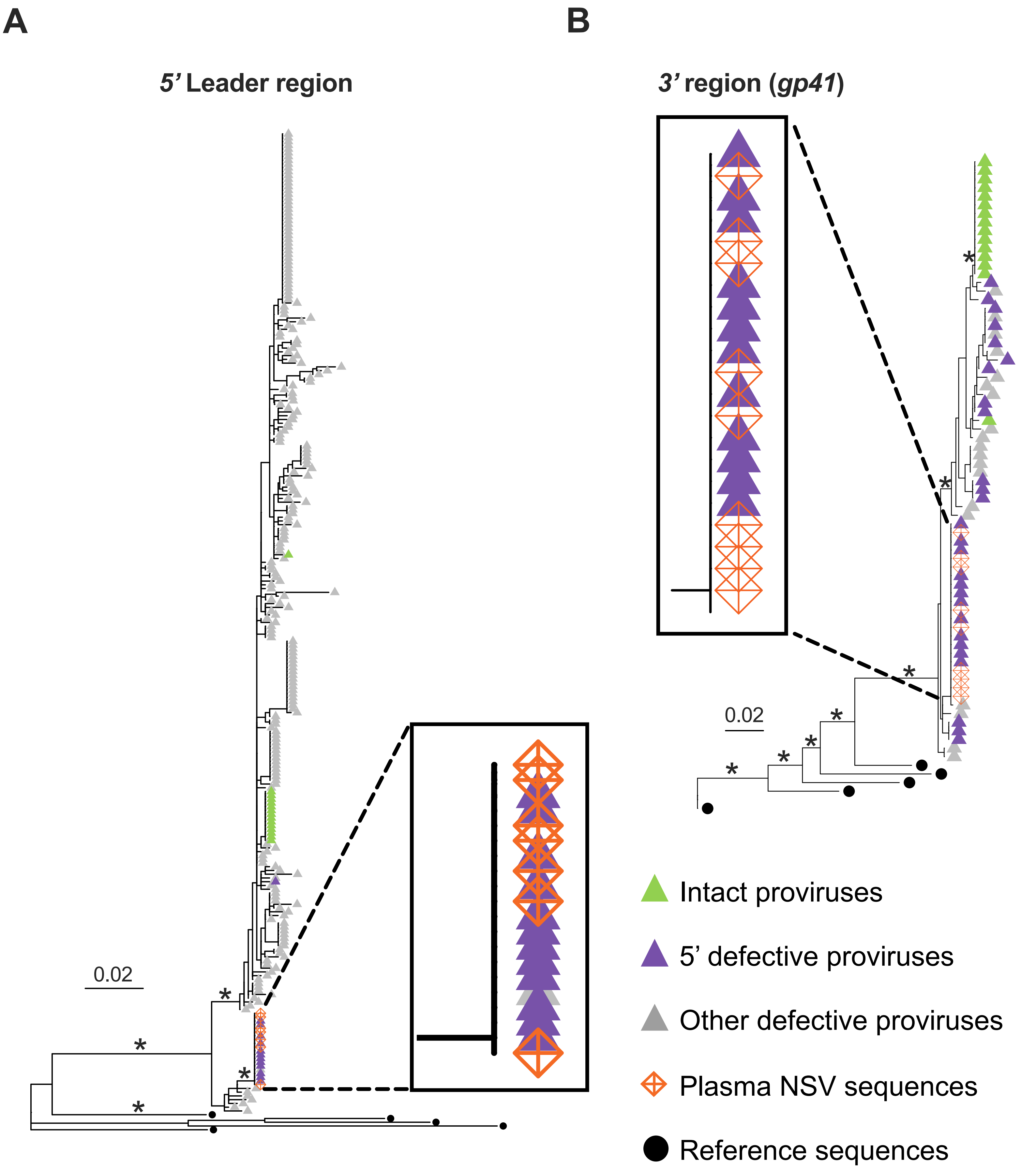
A
5' Leader region
0.02
B
3' region (gp41)
0.02
Intact proviruses
5' defective proviruses
Other defective proviruses
Plasma NSV sequences
Reference sequences

**Figure S2 (previous page). The plasma NSV sequence also matches the provirus clone in the 5' leader and *env* regions.** (A) Maximum likelihood within-host phylogeny inferred from a 545 base-pair region spanning partial 5' leader and *gag* regions, in seven identical plasma sequences isolated during the NSV (orange crossed diamonds) and 255 non-hypermutated proviral sequences containing this region (green, purple and grey triangles denoting intact, 5' defective and other defective proviruses, respectively). A majority of 5' defective sequences had large deletions in this region, so they are not included in this tree. (B) Maximum likelihood within-host phylogeny inferred from a 498 base-pair region covering *gp41*, in nine identical plasma sequences isolated during the NSV and 61 non-hypermutated proviruses containing this region. In both trees, black filled circles denote reference sequences and asterisks identify bootstrap values >80%. Scale in estimated substitutions per nucleotide site.

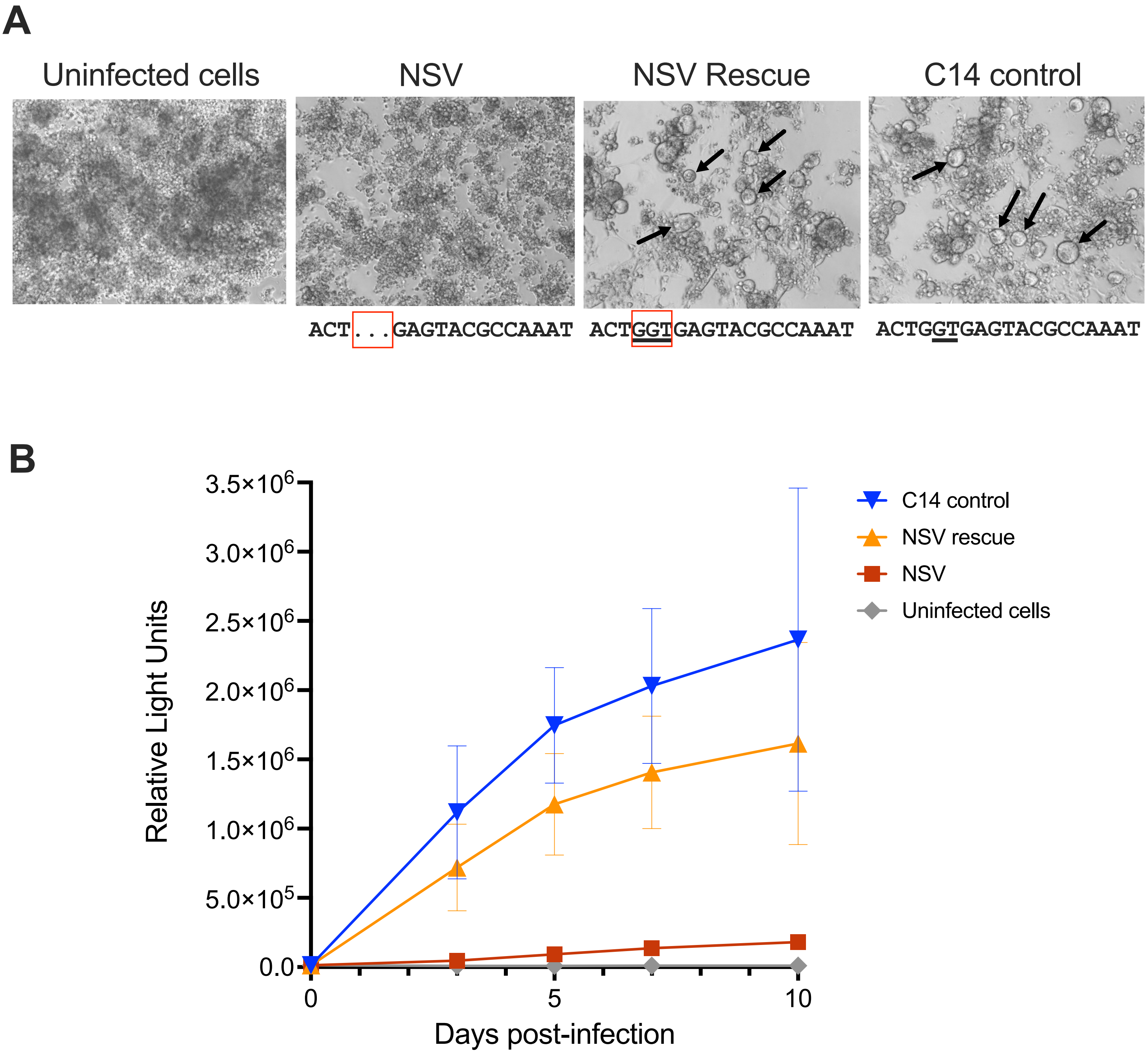


**Figure S3. The NSV virus' replication impairment is confirmed in a co-culture experiment.** (A) Representative microscopy images showing formation of syncytia (examples shown with black arrows) in the NSV-rescue and C14-infected, but not NSV-infected Sup-GGR cells, 10 days after they were co-cultured with HEK293T-cells that had been transfected with equal amounts of each molecular clone. Each virus' MSD region sequence is shown below each image. (B) Viral spread in Sup-GGR cells over 10 days, assessed by quantifying Gaussia luciferase in culture supernatants. Data points and whiskers represent the mean and standard deviation of three technical replicates, respectively.

Figure S4

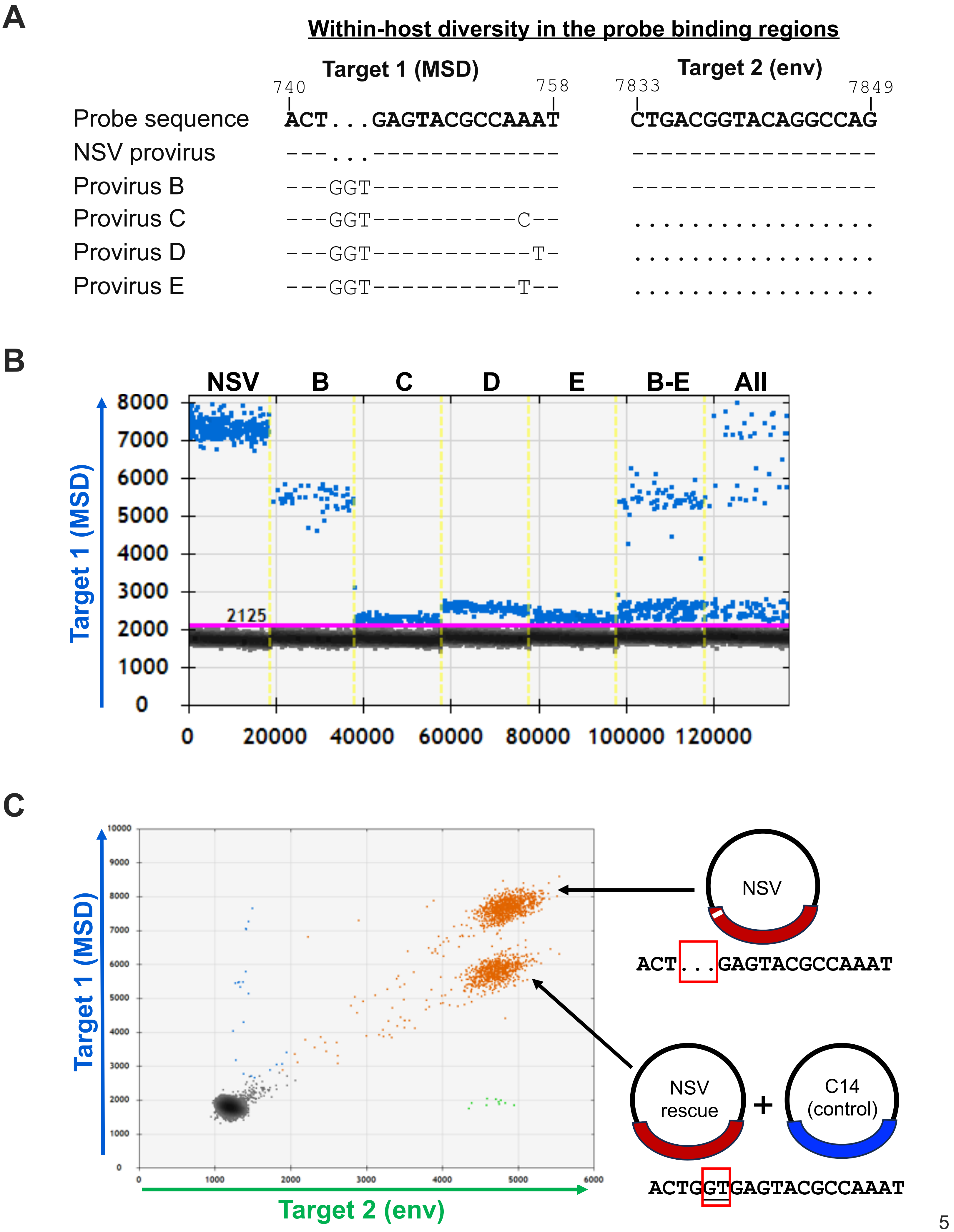
A
Within-host diversity in the probe binding regions
Target 1 (MSD)
Target 2 (env)
740
758
7833
7849
Probe sequence ACT...GAGTACGCCAAAT CTGACGGTACAGGCCAG
NSV provirus ---...------------- -----------------
Provirus B ---GGT------------- -----------------
Provirus C ---GGT----------C-- .................
Provirus D ---GGT-----------T- .................
Provirus E ---GGT----------T-- .................
B
NSV
B
C
D
E
B-E
All
Target 1 (MSD)
2125
0
1000
2000
3000
4000
5000
6000
7000
8000
0
20000
40000
60000
80000
100000
120000
C
Target 1 (MSD)
Target 2 (env)
NSV
ACT...GAGTACGCCAAAT
NSV rescue
+
C14 (control)
ACTGGTGAGTACGCCAAAT

**Figure S4 (previous page). Validation of a custom ddPCR assay to detect the NSV provirus.** Within-host sequence diversity in the assay's MSD (target 1) and *env* (target 2) probe-binding regions, in the NSV provirus and the participant's next four most abundant proviruses (labelled B-E). The target 1 (MSD) probe matched the NSV provirus exactly, while the target 2 (*env*) probe matched essentially all proviruses in this individual. (B) Representative one-dimensional ddPCR plots for target 1 (MSD) when the assay was applied to synthetic templates containing the sequences of the NSV provirus and proviruses B - E, either individually (first five columns) or when pooled (final columns labeled "B-E" and "All"). The NSV sequence consistently yielded signal amplitudes between 7000-8000 units, allowing it to be reliably discriminated from others even in a mixed pool. (C) Representative two-dimensional ddPCR plot when the assay was applied to a mixture of NSV, NSV rescue and C14 HIV molecular clones. Here, the plasmids are detected as double-positive (orange) events, where the NSV provirus is again consistently detected with signal amplitudes between 7000-8000 units for target 1. Single-positive (blue and green) events are due to occasional plasmid shearing.

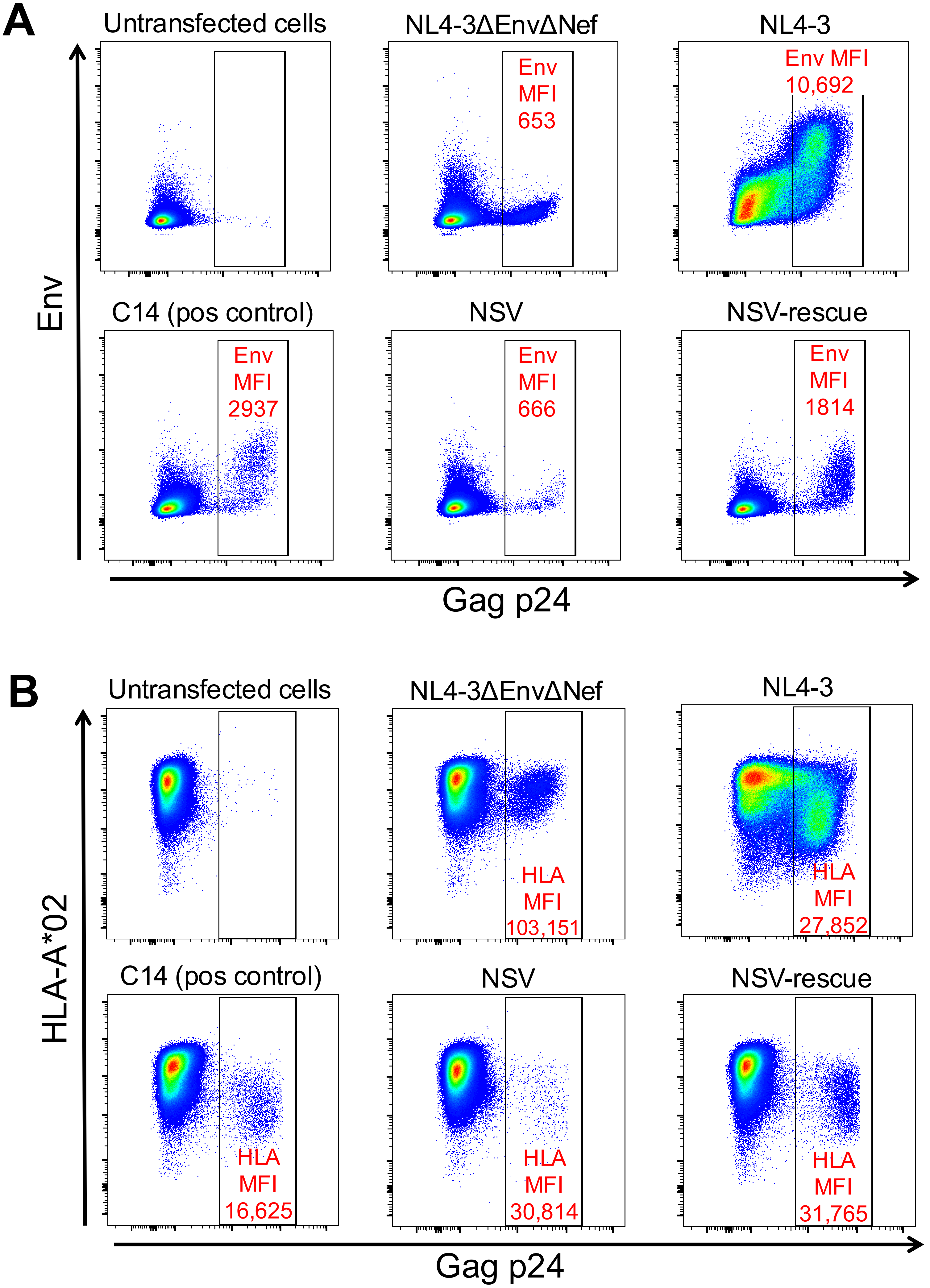
A
Untransfected cells
NL4-3ΔEnvΔNef
Env
MFI
653
NL4-3
Env MFI
10,692
C14 (pos control)
Env
MFI
2937
NSV
Env
MFI
666
NSV-rescue
Env
MFI
1814
Env
Gag p24
B
Untransfected cells
NL4-3ΔEnvΔNef
HLA
MFI
103,151
NL4-3
HLA
MFI
27,852
C14 (pos control)
HLA
MFI
16,625
NSV
HLA
MFI
30,814
NSV-rescue
HLA
MFI
31,765
HLA-A*02
Gag p24

**Figure S5 (previous page). The NSV virus' impaired Envelope expression but preserved HLA downregulation function is confirmed in primary cells.** (A) Representative flow cytometry plots showing intracellular p24 and cell-surface Env expression in CD4+ T-cell-enriched PBMCs 64 hours post-infection with VsV-G-pseudotyped control HIV molecular clone plasmids (top row), and participant molecular clones (bottom row). (B) Representative flow cytometry plots showing intracellular p24 and cell-surface HLA-A*02 expression in the same infected primary cells. This figure shows data from one of two independent experiments. MFI = median fluorescence intensity.

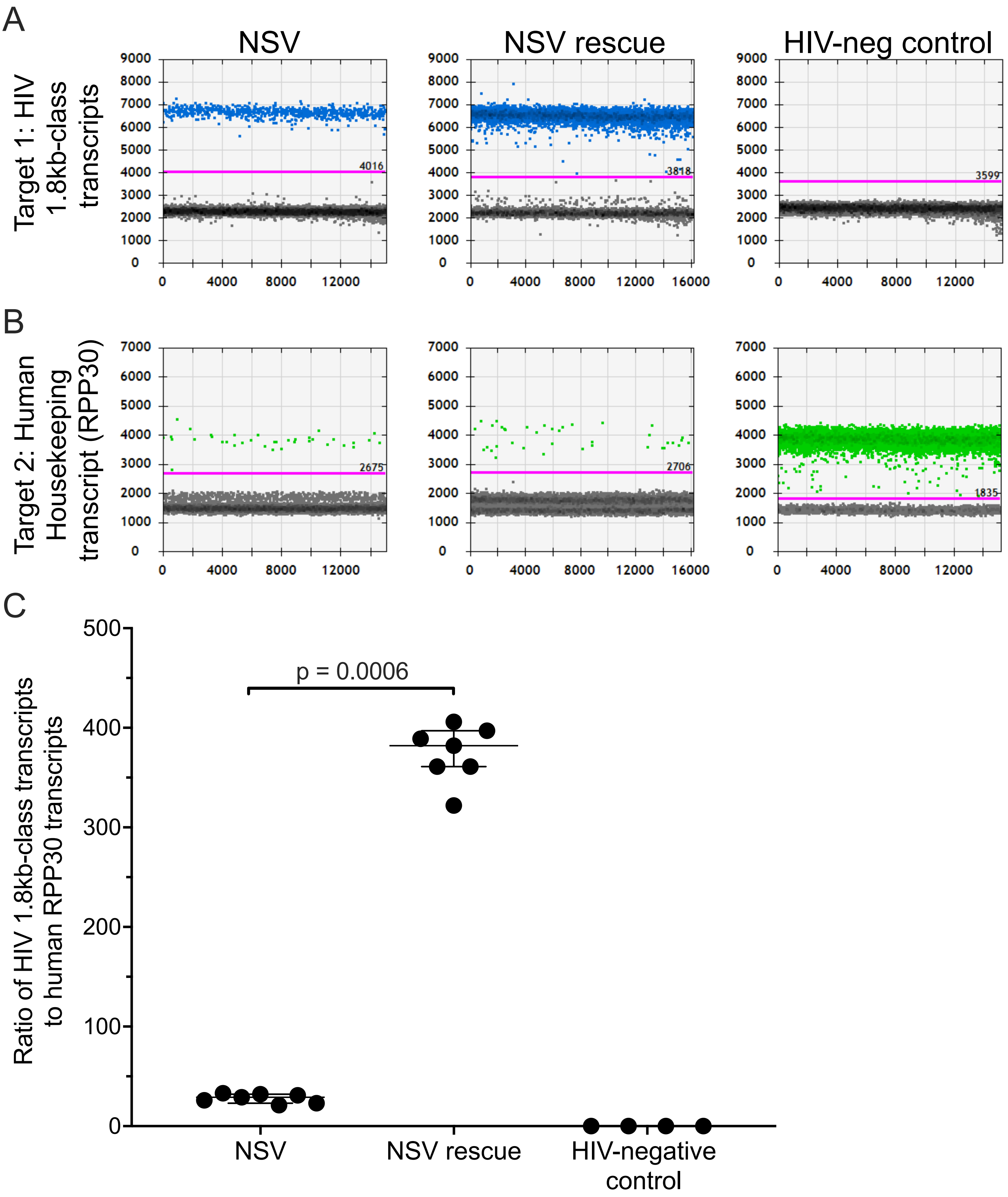
A
NSV
NSV rescue
HIV-neg control
Target 1: HIV 1.8kb-class transcripts
4016
3818
3599
B
Target 2: Human Housekeeping transcript (RPP30)
2675
2706
1835
C
Ratio of HIV 1.8kb-class transcripts to human RPP30 transcripts
p = 0.0006
NSV
NSV rescue
HIV-negative control

**Figure S6 (previous page). The MSD deletion substantially reduces spliced transcript abundance.** (A) Representative ddPCR plots quantifying 1.8kb class HIV transcripts in HEK-293T cells transfected with equal amounts of NSV and NSV rescue plasmids, and in cells from an HIV-negative participant as a control. (B) Representative ddPCR plots quantifying transcripts for the human housekeeping gene RPP30 in these same cells. (C) Ratios of 1.8kb-class HIV transcripts to human RPP30 transcripts for the cell cultures shown in panels A and B, from a minimum of four (maximum six) technical replicates performed per cell type. Statistical significance was assessed using the Mann-Whitney U-test.

Figure S7

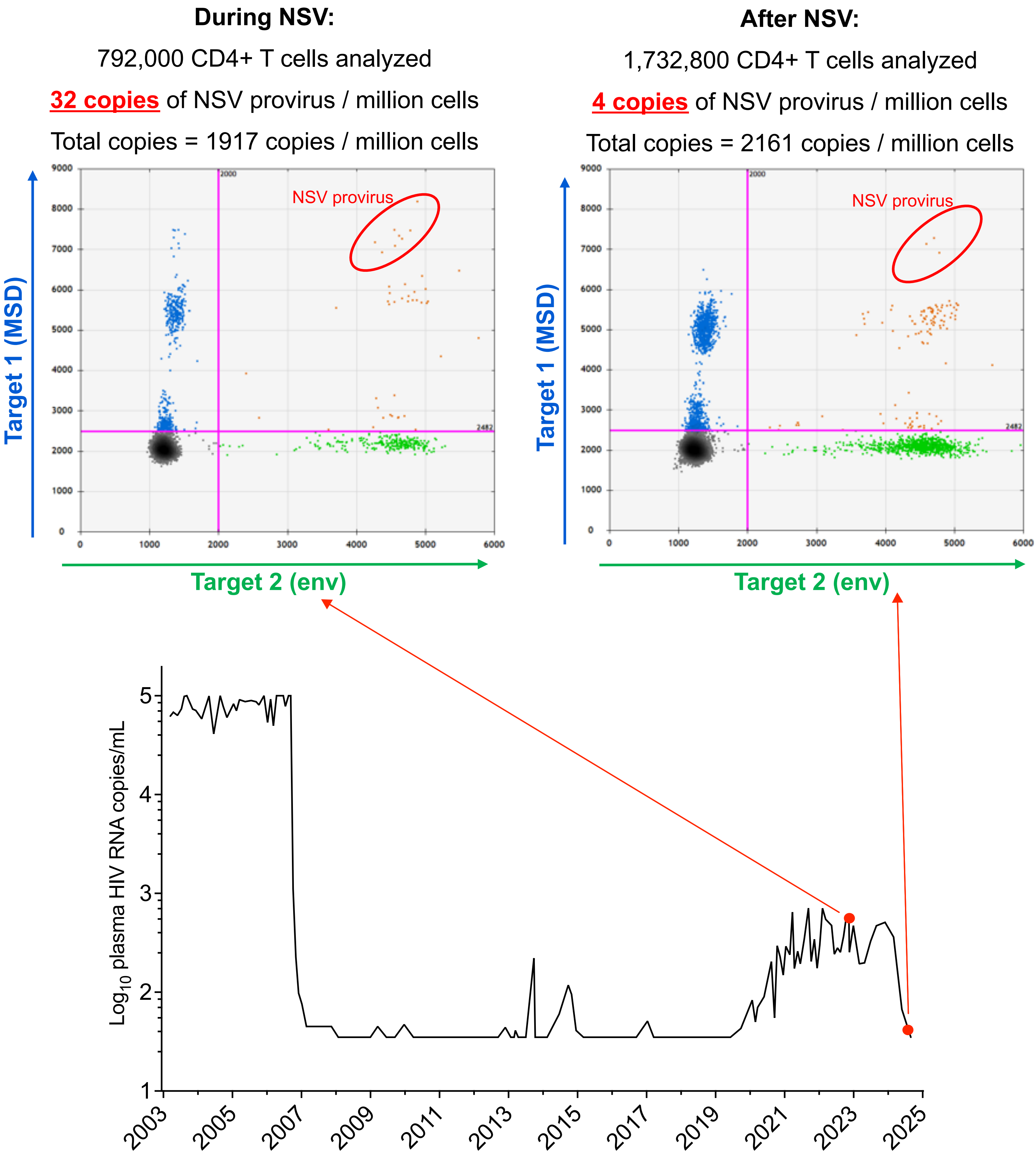
During NSV:
792,000 CD4+ T cells analyzed
32 copies of NSV provirus / million cells
Total copies = 1917 copies / million cells
After NSV:
1,732,800 CD4+ T cells analyzed
4 copies of NSV provirus / million cells
Total copies = 2161 copies / million cells
NSV provirus
NSV provirus
Target 1 (MSD)
Target 1 (MSD)
Target 2 (env)
Target 2 (env)
2000
2482
Log10 plasma HIV RNA copies/mL
2003
2005
2007
2009
2011
2013
2015
2017
2019
2021
2023
2025

**Figure S7 (previous page). Spontaneous resolution of the non-suppressible viremia coincided with contraction of the clonal cell population harboring the NSV provirus.** *Bottom:* The participant's viral load history, with red circles indicating the two time points when blood CD4+ T-cells were analyzed for the presence of the NSV provirus. *Top left:* ddPCR plot showing NSV and total provirus frequency in blood CD4+ T-cells during the viremic period (same data as in Figure 5A). *Top right:* ddPCR plot showing NSV and total provirus frequency in blood CD4+ T-cells after the viremia had resolved.

Figure S8

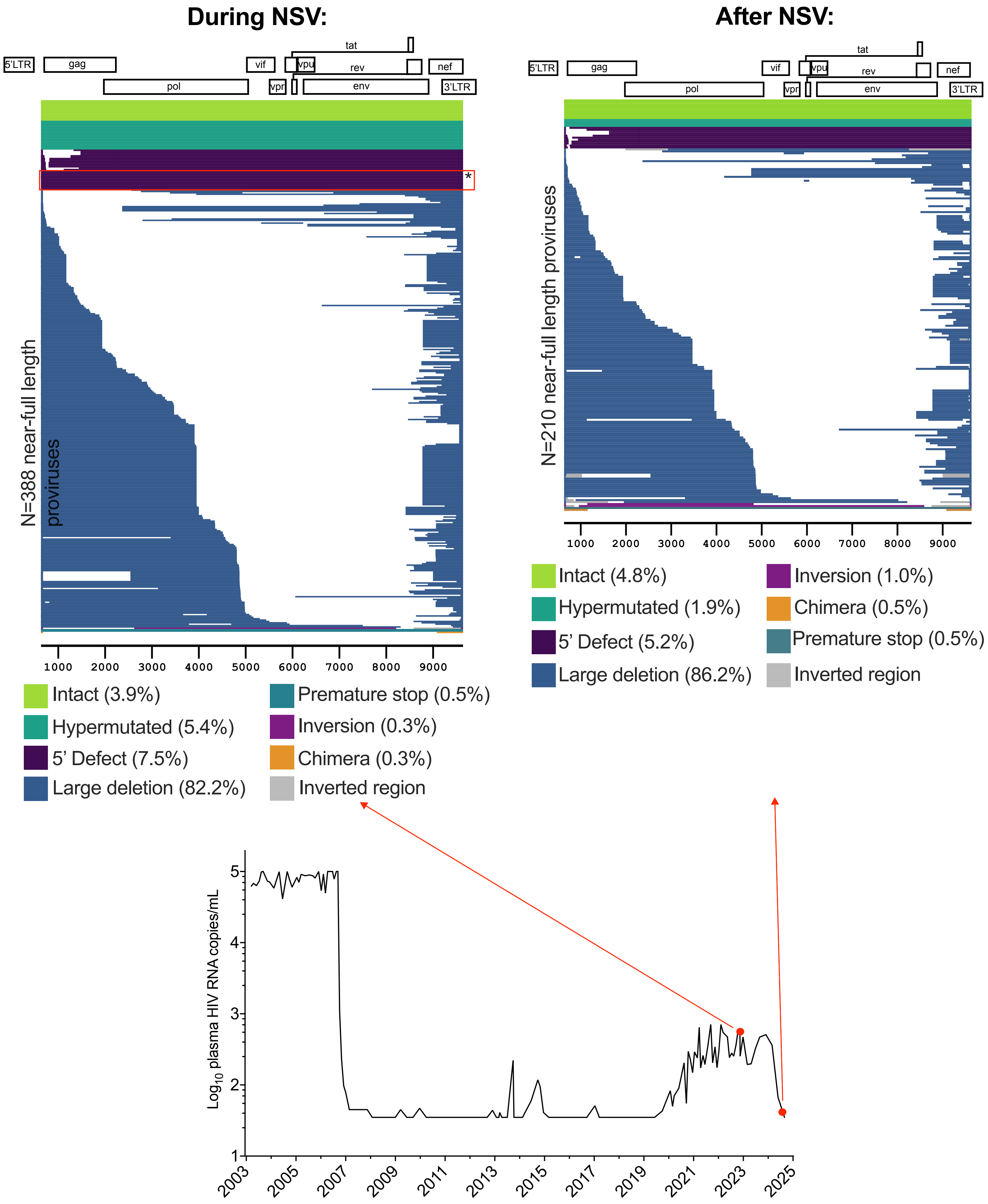

During NSV:
After NSV:
5'LTR
gag
pol
vif
vpr
vpu
tat
rev
env
nef
3'LTR
*
N=388 near-full length proviruses
N=210 near-full length proviruses
1000
2000
3000
4000
5000
6000
7000
8000
9000
Intact (3.9%)
Hypermutated (5.4%)
5' Defect (7.5%)
Large deletion (82.2%)
Premature stop (0.5%)
Inversion (0.3%)
Chimera (0.3%)
Inverted region
Intact (4.8%)
Hypermutated (1.9%)
5' Defect (5.2%)
Large deletion (86.2%)
Inversion (1.0%)
Chimera (0.5%)
Premature stop (0.5%)
Inverted region
Log10 plasma HIV RNA copies/mL
1
2
3
4
5
2003
2005
2007
2009
2011
2013
2015
2017
2019
2021
2023
2025

**Figure S8 (previous page). No substantial change in overall proviral landscape during and after the NSV.** *Bottom:* The participant's viral load history, with red circles indicating the two time points where proviral landscape was characterized in blood CD4+ T-cells. *Top left:* Proviral landscape during NSV (same data as in Figure 1C). The NSV provirus clone is indicated by the red box with the asterisk. *Top right:* Proviral landscape in CD4 +T-cells after after NSV resolution. Proviruses are colored according to genomic integrity (white denotes deletions), with frequencies of each provirus type shown in the figure legend.
