# Supplementary material for: Non-suppressible HIV viremia sustained by clonally expanded CD4+ T cells harboring a genomically defective provirus with an immune-evasive protein expression profile
Source: mBio. 2026 Mar 30;17(5):e03909-25. doi: 10.1128/mbio.03909-25 (PMC13170330; doi:10.1128/mbio.03909-25)
Supplement: Table S1 — Plasma antiretroviral levels measured during nonsuppressible viremia. [file mbio.03909-25-s0002.pdf]

**Table S1: Plasma antiretroviral levels measured during nonsuppressible viremia**

| Sample date | Regimen[a] | Drugs assayed[b] | DRV | COB | DOR | BIC | FTC |
|---|---|---|---|---|---|---|---|
| | | | LLQ-ULQ[c] 40-9000 | LLQ-ULQ[c] 14-3000 | LLQ-ULQ[c] 134-30,000 | LLQ-ULQ[c] 90-20,000 | qualitative (yes/no) |
| | | | UDL cutoff [d] 929 | UDL cutoff [d] 0 | UDL cutoff [d] 147 | UDL cutoff [d] 1690 | |
| Mar 2021 | FTC/TAF/ DRV/COB | DRV, COB, FTC | 3542 | 149 | n/a | n/a | detected |
| Dec 2022 | FTC/TAF/ DRV/COB/ BIC/DOR | DRV, COB, DOR, BIC, FTC | 4422 | 619 | 2264 | 4508 | detected |

[a] FTC=emtricitabine; DRV=darunavir; COB= cobicistat; TAF=tenofovir alafenamide; BIC = bictegravir; DOR=doravirine

[b] Plasma antiretroviral drug levels were assessed by a validated liquid chromatography - tandem mass spectrometry via untimed drug level testing (UDL). The test is called "untimed" because, unlike pharmacokinetic monitoring where samples are assessed at known intervals after drug dosing, dose timing is unknown when testing archived samples. Plasma levels of all non-nucleoside reverse transcriptase inhibitors (NNRTIs), protease inhibitors (PIs), integrase inhibitors and boosting agents are quantified. Plasma levels of some nucleoside analog reverse transcriptase inhibitors (NRTIs) can also be detected qualitatively.

[c] LLQ and ULQ = assay lower and upper limits of quantification for each drug, in ng/mL.

[d] UDL cutoff = In contrast to pharmacokinetic monitoring, which can be used to measure whether drug concentrations are within a therapeutic range at a given time, UDL measurements can only be interpreted qualitatively. Since these tests are untimed, a drug-specific threshold corresponding to the published mean $C_{min}$ or $C_{trough}$ (the lowest plasma concentration during each dosing cycle) for that drug derived from published studies, less one standard deviation, is used to define whether drug is present in plasma, or whether drug levels are "low/absent". In clinical testing, results are reported qualitatively (drug detected vs. low/absent), but actual values are reported here, in ng/mL. Plasma drug levels for all tested drugs were well above UDL cut-offs, supporting adequate drug adherence, absorption, distribution, metabolism and excretion.
