# Supplementary material for: Non-suppressible HIV viremia sustained by clonally expanded CD4+ T cells harboring a genomically defective provirus with an immune-evasive protein expression profile
Source: mBio. 2026 Mar 30;17(5):e03909-25. doi: 10.1128/mbio.03909-25 (PMC13170330; doi:10.1128/mbio.03909-25)
Supplement: Table S2 — Primers and probes. [file mbio.03909-25-s0003.pdf]

Table S2 - List of primers and probes

Assay	PCR reaction	Primer name	Direction	HXB2 coordinates	Sequence (5' to 3')
Near full-length amplification	Outer	623-Fi(gag)	Forward	623 - 629	AAATCTCTAGCAGTGGCGCCCGAACAG
		R9662-9686	Reverse	9686 - 9662	TGAGGGATCTCTAGTTACCAGAGTC
	Nested	U5-638F	Forward	638 - 666	GCGCCCGAACAGGGACYTGAAARCGAAAG
		U5-547R	Reverse	9632 - 9604	GCACTCAAGGCAAGCTTTATTGAGGCTTA
5' amplification	cDNA	GS3R	Reverse	1841 - 1817	TGACATGCTGTCATCATTTCTTCTA
	Outer	623-Fi(gag)	Forward	623 - 649	AAATCTCTAGCAGTGGCGCCCGAACAG
		GS3R	Reverse	1841 - 1817	TGACATGCTGTCATCATTTCTTCTA
	Nested	638-666	Forward	638 - 666	GCGCCCGAACAGGGACYTGAAARCGAAAG
		GS1B	Reverse	1339 - 1316	AATCTTGTGGGGTGGCTCCTTCTG
Pol amplification	cDNA	RT3.1	Reverse	3859 - 3831	GCTCCTACTATGGGTTCTTTCTCTAACTGG
	Outer	5'CP1	Forward	1979 - 2005	GAAGGGCACACAGCCAGAAATTGCAGGG
		RT3.1	Reverse	3859 - 3831	GCTCCTACTATGGGTTCTTTCTCTAACTGG
	Nested	2.5	Forward	2011 - 2039	CCTAGGAAAAAGGGCTGTTGGAAATGTGG
		RT3798R	Reverse	3798 - 3777	CAAACTCCCACTCAGGAATCCA
3' amplification	cDNA	GP41RO	Reverse	8819 - 8797	CTTTTTGACCACTTGCCACCCAT
	Outer	GP41Fo	Forward	7626 - 7648	TTCAGACCTGGAGGAGGAGATAT
		GP41RO	Reverse	8819 - 8797	CTTTTTGACCACTTGCCACCCAT
	Nested	GP41Fi	Forward	7652 - 7674	GGACAATTGGAGAAGTGAATTAT
		GP41Ri	Reverse	8771 - 8749	CTGTCTTATTCTTCTAGGTATGT
LTR amplification	Outer	Auto FwdLTR	Forward	1 - 27	TGGATGGGTTAATTTACTCCARGAA
		Auto Rev2Gag	Reverse	1386 - 1367	TTGCATAGCTGCCTGGTGTC
	Nested	Auto Fwd1LTR	Forward	5 – 29	TGGGTTAATTTACTCCARGAAAAGA
		Auto RevGag	Reverse	1317 - 1294	TGATAGTGCTGTGAACATGGGTAT
HIV-1 spliced Transcripts: 4 kb class	cDNA	Auto-4REV-OUT	Reverse	6101 - 6072	CTACTAGTCCTACTATTGCAGATATAACTA
	Outer	623-Fi(gag)	Forward	623 - 649	AAATCTCTAGCAGTGGCGCCCGAACAG
		Auto -4REV-OUT	Reverse	6101 - 6072	CTACTAGTCCTACTATTGCAGATATAACTA
	Nested	U5-638F	Forward	638 - 666	GCGCCCGAACAGGGACYTGAAARCGAAAG
		Auto -4REV-IN	Reverse	6077 - 6046	TAACTAAAGGTTGCATCACATATACTAATTAT

HIV-1 spliced Transcripts: 1.8 kb class	cDNA	Auto -1.8REV-OUT	Reverse	5969 - 5947	TGGTGATGGGTAAGGGTTGCTTT
	Outer	623-Fi(gag)	Forward	623 - 649	AAATCTCTAGCAGTGGCGCCCGAACAG
		Auto -1.8REV-OUT	Reverse	5969 - 5947	TGGTGATGGGTAAGGGTTGCTTT
	Nested	U5-638F	Forward	638 - 666	GCGCCCGAACAGGGACYTGAAARCGAAAG
		Auto -1.8REV-IN	Reverse	1668 - 1643	TAAGGGTTGCTTTGGTACAGGATTTT
HIV-1 spliced Transcripts: 1 kb class	cDNA	EIR	Reverse	9043 - 9015	CCTTGTAAGTCATTGGTCTTAAAGGTACC
	Outer	623-Fi(gag)	Forward	623 - 649	AAATCTCTAGCAGTGGCGCCCGAACAG
		EIR	Reverse	9043 - 9015	CCTTGTAAGTCATTGGTCTTAAAGGTACC
	Nested	U5-638F	Forward	638 - 666	GCGCCCGAACAGGGACYTGAAARCGAAAG
		GP41RO	Reverse	8819 - 8797	CTTTTTGACCACTTGCCACCCAT
NSV provirus detection (ddPCR)	RPP30 (human)	RPP30Fwd	Forward	NA	GATTTGGACCTGCGAGCG
	RPP30 (human)	RPP30Probe	NA (probe)	NA	VIC[a]-CTGACCTGAAGGCTCT-MGBNFQ[b]
	RPP30 (human)	RPP30Rev	Reverse	NA	GCGGCTGTCTCCACAAGT
	RPP30-Shear (human)	ShearFwd	Forward	NA	CCATTTGCTGCTCCTTGGG
	RPP30-Shear (human)	ShearProbe	NA (probe)	NA	6-FAM[c]-AAGGAGCAAGGTTCTATTGTAG-MGBNFQ
	RPP30-Shear (human)	ShearRev	Reverse	NA	CATGCAAAGGAGGAAGCCG
	HIV MSD	GagFwd	Forward	692 - 711	CAGGACTCGGCTTGCTGAAG
	HIV MSD	GagProbe	NA (probe)	743 - 761	6-FAM-ACTGAGTACGCCAAATTTT-MGBNFQ
	HIV MSD	GagRev	Reverse	797 - 775	GCACCCATCTCTCTCCTTCTAGC
	HIV env	EnvFwd	Forward	7809 - 7825	ACTATGGGCGCGGCGTC
	HIV env	EnvProbe	NA (probe)	7849 - 7833	VIC-CTGGCCTGTACCGTCAG-MGBNFQ
	HIV env	EnvRev	Reverse	7939 - 7921	CCCCAGACCGTGAGTTTCA
	HIV env	HypermutationProbe	NA (probe)	7781 - 7798	**CCTTAGGTTCTTAGGAGC-MGBNFQ**
HIV spliced transcript quantification (ddPCR)	HIV 1.8 kb spliced	Auto_MSFwd	Forward	5978 - 5996	AAGAAGCGGAGACAGCGAC
	HIV 1.8 kb spliced	Auto_MSProbe	NA (probe)	6040 - 6045/ 8379 -8391[d]	6-FAM-AAAGCAACCCTTACCCATC-MGBNFQ
	HIV 1.8 kb spliced	Auto_MSRev	Reverse	8475 - 8456	**CGGTCACTAATCGAGTGGAT**

	Housekeeping transcript	RPP30Fwd	Forward	NA	GATTTGGACCTGCGAGCG
	Housekeeping transcript	RPP30Probe	NA (probe)	NA	VIC-CTGACCTGAAGGCTCT-MGBNFQ
	Housekeeping transcript	RPP30Rev	Reverse	NA	GCGGCTGTCTCCACAAGT
MIP-Seq step 1: Whole human genome amplification by Multiple Displacement Amplification (MDA) from provirus endpoint-diluted DNA	MDA	P7R9706	NA	various	/5Phos/CCACA*C*T[e]
	MDA	P7R9523	NA	various	/5Phos/CAGCT*G*C
	MDA	P7R6447	NA	various	/5Phos/CACAG*G*C
	MDA	P7R5041R	NA	various	/5Phos/CATCT*G*T
	MDA	P7RCD4R	NA	various	/5Phos/CTTCT*C*C
	MDA	P7R707	NA	various	/5Phos/CAAGC*C*G
	MDA	P7R9686	NA	various	/5Phos/GGATC*T*C
	MDA	P7RCP2	NA	various	/5Phos/GTTTA*A*C
	MDA	P6N (random hexamer)	NA	various	/5Phos/NNNN*N*N
MIP-seq linear extension for integration site	Linear Extension	GS1R	Reverse	1097-1074	TTATCTAAAGCTTCCTTGGTGTCT
	Linear Extension	GS1B	Reverse	1339-1316	AATCTTGTGGGGTGGCTCCTTCTG
Inverse PCR for integration site determination	Inv. PCR outer	Out_FOR(964-990)	Forward	964-990	CAGCAGAAGGATGTCAGCAAATAATGA
	Inv. PCR outer	Out_REV(838-864)	Reverse	864-838	GAATCCCTTCCCATCTATCTAATTGTC
	Inv. PCR inner	In_FOR(1174-1196)	Forward	1174-1196	GGAAACAGCAGCACGGTCAGTCAC
	Inv. PCR inner	In_REV(760-788)	Reverse	788-760	/5Phos/CGCTAGTCAAAATTTGGCGTACTC*A*G[e]

[a]VIC = 2′-chloro-7′phenyl-1,4-dichloro-6-carboxy-fluorescein
[b]MGBNFQ = *3'* Minor groove binder; NFQ = Nonfluorescent quencher
[c]FAM = carboxyfluorescein
[d]Probe spans the D4/A7 junction of 1.8 kb multiply spliced transcripts
[e]The 5' terminus of the MDA primers are phosphorylated (indicated by /5Phos/) and the terminal and 3'-teminal positions contain phosphorothioate linkages (indicated by * to the left of the base) to protect from 3' exonuclease activity
